# Chlorine‐Functionalized Silane‐Modified Copper Electrocatalyst for Enhanced CO_2_ Reduction to Multi‐Carbon Products

**DOI:** 10.1002/advs.76741

**Published:** 2026-07-24

**Authors:** Ying Ying Ch'ng, Sankhadip Saha, Ming Zhang, Shujie Zhou, Lixue Jiang, Putri Ramadhany, Jitraporn Vongsvivut, Adrian Cernescu, Priyank Vijaya Kumar, Yihao Shan, Zhipeng Ma, Xiaoxuan Luo, Rahman Daiyan, Zhaojun Han, Rose Amal

**Affiliations:** ^1^ School of Chemical Engineering The University of New South Wales Sydney New South Wales Australia; ^2^ Infrared Microspectroscopy (IRM) Beamline ANSTO—Australian Synchrotron Clayton Victoria Australia; ^3^ Attocube systems GmbH Munich‐Haar Germany; ^4^ School of Minerals and Energy Resources Engineering The University of New South Wales Sydney New South Wales Australia; ^5^ Eastern Institute of Technology Ningbo Zhejiang China

**Keywords:** C_2+_, carbon dioxide reduction, silane

## Abstract

Electrochemical CO_2_ reduction reaction (CO_2_RR) on Cu‐based catalysts toward multi‐carbon (C_2+_) products remains challenging due to sluggish CO_2_ activation, high C–C dimerization energy barriers, and competing hydrogen evolution reaction. While halide species, such as chlorine, promote CO_2_ activation, and silane‐based modifiers can regulate charge transfer to enhance C–C dimerization, these effects are typically decoupled when applied separately. Herein, we identify 3‐chloropropyltrimethoxysilane (CPTMS) as a molecular modifier that integrates halide functionality and silane anchoring within one scaffold, enabling spatially coupled regulation of CO_2_RR intermediates at the Cu interface. Introduced via a one‐step electrodeposition strategy, the CPTMS‐modified Cu catalyst exhibits a 5‐fold enhancement in C_2+_ faradaic efficiency, reaching 75% ± 2% compared with 15% ± 4% for unmodified CuO*_x_* at −1.5 V vs. the reverse hydrogen electrode with a partial current density of 105 mA cm^−2^. In‐situ synchrotron‐based Fourier transform infrared reveals that the spatially coupled chlorine and silicon functionalities sequentially optimize the CO_2_RR pathway by promoting CO_2_ activation, *COOH formation, *CO protonation, and subsequent C–C dimerization on Cu active sites achieving enhanced C_2+_ product formation. This work elucidates molecularly integrated halide‐silane functionalization‐enabled synergistic interfacial control, offering a rational design strategy for advancing selective C_2+_ product formation in CO_2_RR.

## Graphical abstract

A chlorine‐functionalized silane modifier (CPTMS) is introduced to cooperatively regulate CO_2_ reduction to C_2+_ products on Cu‐based catalysts. Dual‐functional interface sequentially enhances CO_2_ activation, intermediates protonation and C─C coupling, delivering a 5‐fold increase in C_2+_ faradaic efficiency (FE, 75% vs. 15%) in CuO_x_CPTMS, while suppressing the hydrogen evolution reaction through interfacial microenvironment engineering.

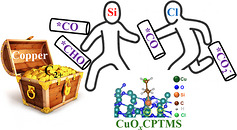

## 1 Introduction

Leveraging the increased waste anthropogenic carbon dioxide (CO_2_) has been posited as a compelling pathway in mitigating climate change. Specifically, electrochemical CO_2_ reduction reaction (CO_2_RR) powered by renewable electricity emerges as a green technology in converting CO_2_ to valuable chemicals under ambient conditions [1]. CO_2_RR is particularly attractive for producing energy‐dense multi‐carbon (C_2+_) products such as ethylene as the plastic precursor [2] and ethanol with a diverse application portfolio [3, 4]. Among the electrocatalysts explored, copper (Cu) has shown its unique capability with optimum binding strengths, enabling efficient CO_2_ adsorption, activation, and subsequent stabilization of intermediates that promote C─C dimerization toward C_2+_ products formation [5]. Nevertheless, C_2+_ product selectivity of Cu‐based catalysts still remains unsatisfactory, challenged by sluggish CO_2_ activation and high C─C dimerization energy barriers, aside from the competition from the hydrogen evolution reaction (HER) [6]. To overcome these challenges, it is necessary not only to improve the intrinsic activity of Cu active sites but also to precisely regulate the interfacial reaction environment.

In this context, halides have been widely reported to tune the catalytic microenvironment by inducing localized pseudo‐positivity charge on Cu (Cu^δ+^) through interfacial electronic interactions [7], thereby enhancing CO_2_ activation and increasing the current density (*j*) [8, 9]. In addition to the CO_2_ activation, C─C dimerization is a selectivity‐determining step for C_2+_ products, which is governed by the kinetics of proton‐coupled electron transfer (PCET) [10, 11]. Various catalyst design strategies have been explored to optimize the C─C dimerization step such as facet engineering, oxygen vacancy‐associated defect engineering, and microenvironment modulation [12, 13, 14]. More recently, molecular functionalization has emerged as an effective approach to optimize intermediate adsorption at the Cu interface. Particularly, silicon‐modified Cu catalysts have been demonstrated to improve C_2+_ products by enhancing *CO protonation, stabilizing protonated intermediates of *CHO, thus reducing C─C dimerization energy barriers through *CO─*CHO coupling [15]. For instance, Yan et al. demonstrated that Cu─O─Si catalyst can shift the protonation kinetics from monocarbon product formation on a pristine catalyst toward ethylene production [16]. Furthermore, silicon surface modification by using silane was reported to induce superhydrophobicity on the Au/C electrode, which enhanced CO_2_RR performance by reshaping a stable gas‐liquid‐solid interface [17]. Despite these advances, halide and molecular functionalization are typically introduced separately (Table S1), leading to spatially decoupled regulation of electronic structure and interfacial engineering, which limits their cooperative impact on multistep CO_2_RR pathways.

To address this limitation, an integrated molecular strategy that enables spatially coupled halides and molecular functionalization is highly desirable. Here, we identify 3‐chloropropyltrimethoxysilane (CPTMS) as a multifunctional molecular modifier that unifies halide functionality and silane anchoring within a single molecular scaffold. In this design, the chlorine moiety is crafted to promote CO_2_ activation, while the silane group chemically anchors to CuO*_x_* via Si─O─Cu linkages, regulating charge transfer, and stabilizing key *CO‐derived intermediates. Simultaneously, the alkylsilane framework introduces localized hydrophobicity that suppresses HER, collectively enabling cooperative and spatially coupled interfacial control beyond independent halide or silane modification. We demonstrate CPTMS‐modified CuO*_x_* via a one‐step electrodeposition method for efficient CO_2_RR into C_2+_ products. The individual contributions of Cl and Si are systematically studied. Our investigation shows the influence of Cl in shifting the *d*‐band center closer to the Fermi level thereby facilitating the activation of CO_2_, while incorporation of Si improves interfacial charge transfer, leading to enhanced *CO protonation. Moreover, mechanistic studies indicate that the combined roles of Cl and Si enable a sequential optimization of the CO_2_RR pathway, spanning CO_2_ activation, *COOH formation, *CO protonation to C─C dimerization on Cu active sites. Notably, CuO*_x_* CPTMS achieved a 5‐fold increment in total faradaic efficiency of C_2+_ products (FE_C2+_) compared with CuO*_x_* (75% vs. 15%) at −1.5 V vs. reversible hydrogen electrode (V_RHE_). These findings elucidate how integrated halide‐silane molecular functionalization enables synergistic interfacial modification, providing a rational catalyst design paradigm for advancing selective C_2+_ product formation in CO_2_RR.

## 2 Results and Discussion

### 2.1 Catalysts Preparation and Characterization

CuO*_x_* catalyst and CPTMS‐modified CuO*_x_* catalyst (CuO*_x_* CPTMS) (Figure S1a) were synthesized via the electrodeposition method. To decouple the individual roles of Si and Cl in the modification of CuO*_x_* catalyst, Cl‐CuO*_x_*, and CuO*_x_* PTMS were synthesized as control samples by using potassium chloride (KCl) and propyltrimethoxysilane (PTMS; Figure S1b), respectively.

**FIGURE 1:**
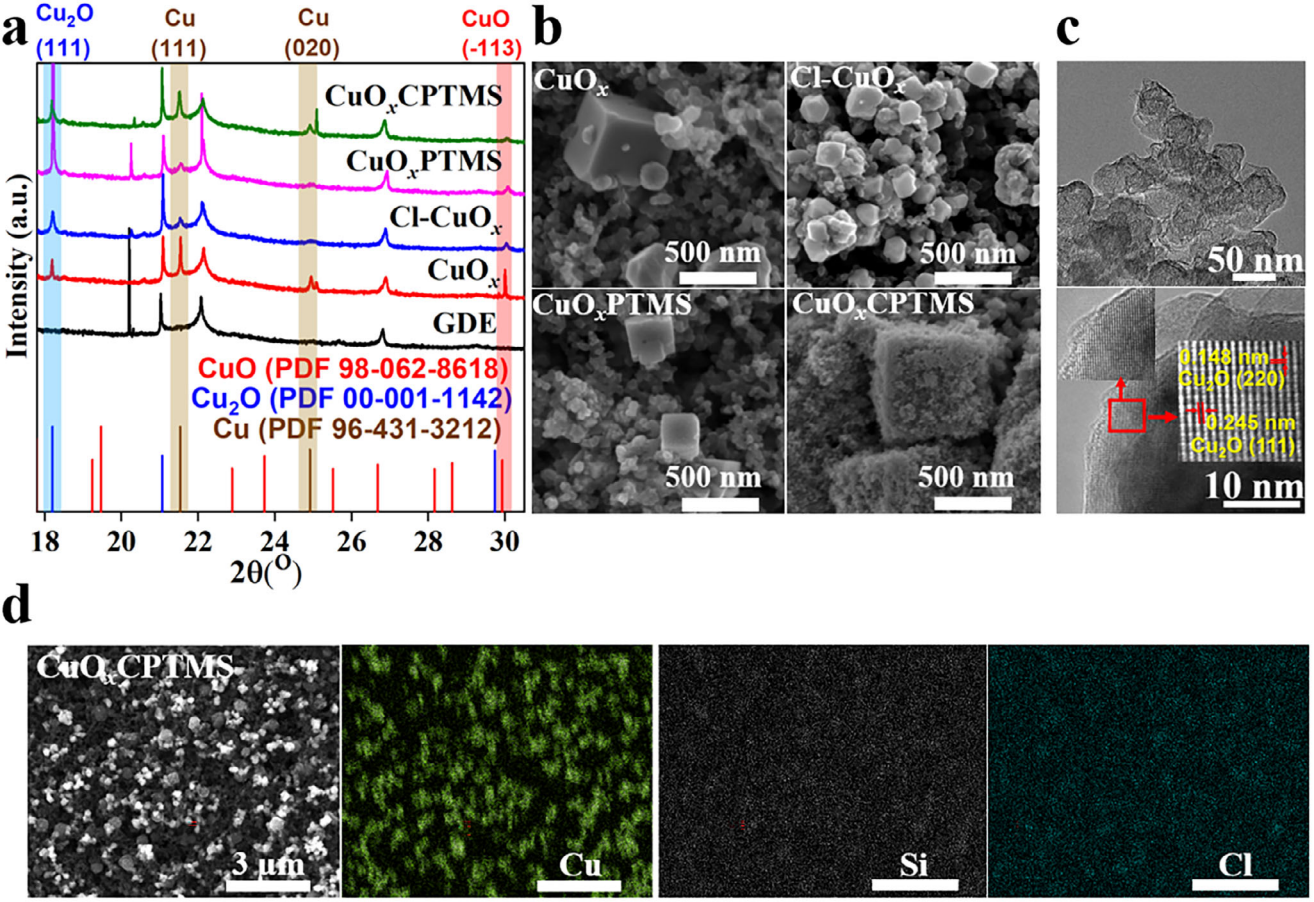
(a) Synchrotron radiation‐based PD (at 0.7748 Å) patterns of CuO*_x_*, Cl‐CuO*_x_*, CuO*_x_* PTMS, and CuO*_x_* CPTMS, (b) SEM images of CuO*_x_*, Cl‐CuO*_x_*, CuO*_x_* PTMS, and CuO*_x_* CPTMS, (c) TEM image of CuO*_x_* CPTMS at 50 nm scale and 10 nm scale with lattice fringes. Inset shows the lattice spacing of the probed area, (d) SEM‐EDS mapping on CuO*_x_* CPTMS.

Following the modifications of CuO*_x_* catalysts with Si, Cl, or Si─Cl functional groups, the bulk crystallinity of the as‐prepared catalysts was characterized via synchrotron radiation‐based ex‐situ X‐ray powder diffraction (SR‐XRPD) in Figure 1a. The diffraction peaks of all as‐prepared catalysts at 2θ = 21.5°, 24.9° are well indexed to (111) and (020) of phases from the standard metallic Cu crystal structure (PDF: 96‐431‐3212). Another main diffraction peak of all catalysts is observed at 2θ = 18.2°, which can be assigned to the (111) phase of the standard crystal structure of Cu_2_O (PDF: 00‐001‐1142). The diffraction peak of CuO (‐113) is detected at 2θ ≈ 30.0° (PDF: 98‐0.62‐8618), evidencing the presence of crystalline CuO, Cu_2_O, and Cu in these catalysts. The difference in the intensity of the exposed facets between these catalysts and CuO*_x_* is likely due to the variation in the nucleation process during synthesis when the electrodeposition bath consists of modifiers [18]. On the other hand, the absence of diffraction peaks associated with Si, Cl, or Si─Cl related crystal phases indicates that no additional crystalline structures are formed through self‐nucleation during catalyst electrodeposition.

The morphology of catalysts was examined by scanning electron microscopy (SEM) in Figure 1b. CuO*_x_*, Cl‐CuO*_x_*, and CuO*_x_* PTMS consist of irregularly shaped and sized particles mixed with cuboid‐like particles whereas CuO*_x_* CPTMS exhibited a predominantly cuboid‐like morphology, albeit with a relatively rough surface. To further verify the impact of CPTMS on the morphology of the catalyst, transmission electron microscopy (TEM) was employed. At the scale of 50 nm (Figure 1c), the nanocuboid‐like feature of CuO*_x_* CPTMS is distinguishable, which confirms the survey from SEM. Additionally, the examined particle from CuO*_x_* CPTMS exhibits lattice fringes at *d*‐spacing of 0.245 and 0.148 nm, which can be assigned to Cu_2_O (111) and Cu_2_O (220) facets, respectively (Figure 1c). The presence of Cu_2_O (111) disclosed by the TEM image is consistent with the observation from *ex‐situ* SR‐XRPD in Figure 1a. Additionally, the observed morphology of CuO*_x_* from the SEM image is confirmed with the survey with TEM (Figure S2a), and the presence of Cu_2_O (111) and CuO (021) was revealed by the lattice fringes at *d*‐spacing of 0.245 and 0.162 nm, respectively (Figure S2b). The element composition of CuO*_x_* CPTMS (Figure S3) was further ascertained in energy dispersive X‐ray spectroscopy (EDS), revealing a uniform distribution of Si and Cl on particles of CuO*_x_* CPTMS (Figure 1d).

**FIGURE 2:**
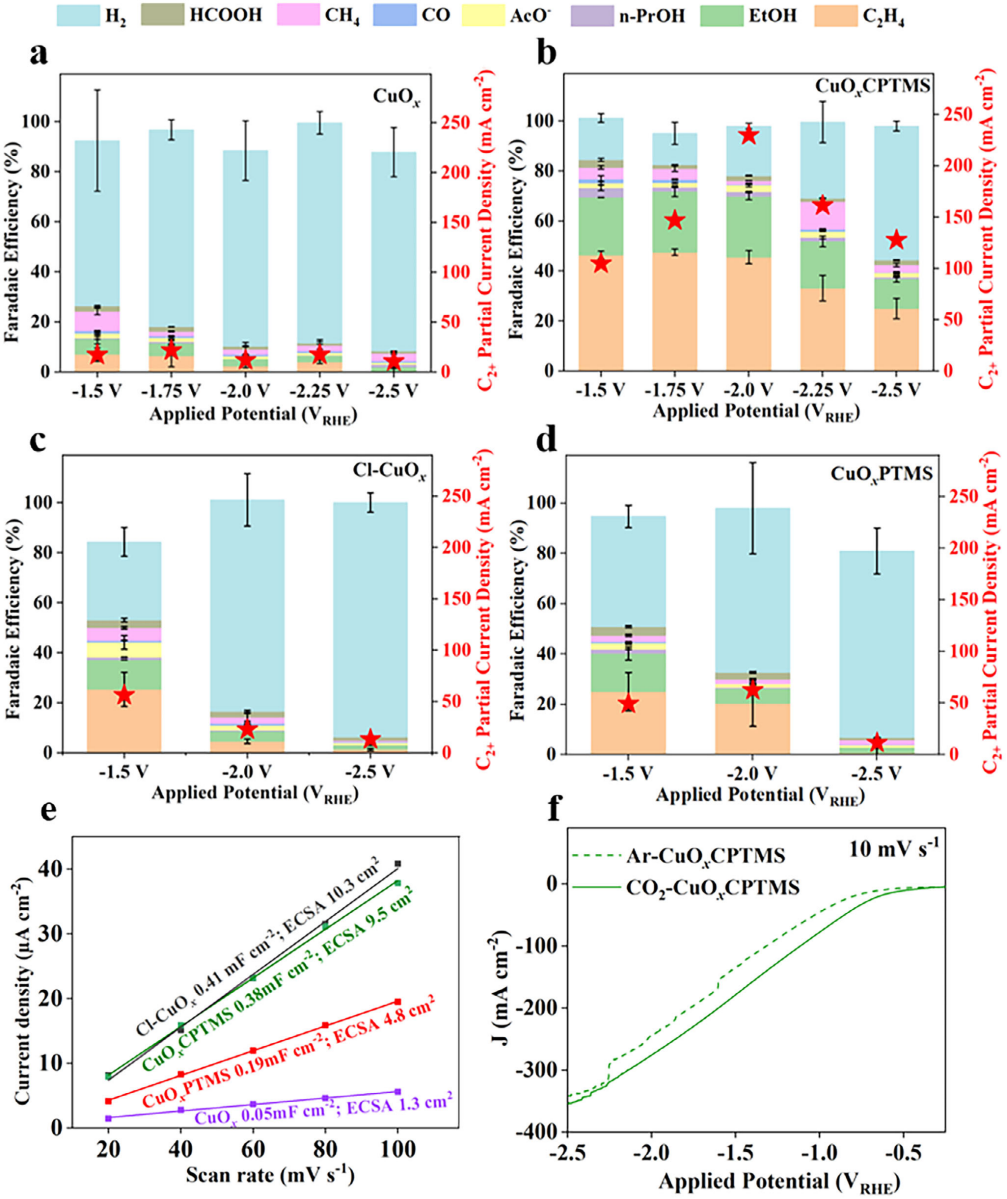
Faradaic efficiency and the associated C_2+_ products partial current density during CO_2_RR in 1 m KOH of (a) CuO*_x_*, (b) CuO*_x_* CPTMS, (c) Cl‐CuO*_x_*, (d) CuO*_x_* PTMS, (e) ECSA of of CuO*_x_*, Cl‐CuO*_x_*, CuO*_x_* PTMS, and CuO*_x_* CPTMS, and iR drop uncompensated LSV in Ar and CO_2_ environments of (f) Cuo*_x_* CPTMS.

Subsequently, the potential‐dependent CO_2_RR performance of CuO*_x_* and CuO*_x_* CPTMS was evaluated using a flow‐cell (Figure S4) in a 1 m KOH aqueous electrolyte. The gas and liquid products were quantified by offline gas chromatography (GC) and offline ^1^H nuclear magnetic resonance (NMR), respectively. CO_2_RR activity was measured at fixed potentials from −1.5 V_RHE_ to −2.5 V_RHE_, with each potential held for 10 min. The detected products included C_2_H_4_, C_2_H_5_OH (EtOH), propanol (PrOH), formate (HCOOH), carbon monoxide (CO), acetate (AcO^−^), and methane (CH_4_), aside from H_2_ from HER (Figure 2a,b). CuO*_x_* shows optimum CO_2_RR performance at −1.5 V_RHE_ where the FE_C2+_ was 15% with Faradaic efficiency of H_2_ (FE_H2_) at 66%, which corresponded to C_2+_ partial current density (*j*_C2+_) of 17 mA cm^−2^ and H_2_ partial current density (*j*_H2_) of 76 mA cm^−2^ (Figure S5a). In contrast, CuO*_x_* CPTMS recorded a peak FE_C2+_ of 75% (*j*_C2+_ = 145 mA cm^−2^) and FE_H2_ was at 13% (*j*_H2_ = 24 mA cm^−2^) at −1.75 V_RHE_. At −1.5 V_RHE_, CuO*_x_* CPTMS recorded FE_C2+_ of 75% albeit at a *j*_C2+_ of 105 mA cm^−2^. Further increase of negative applied potential at ‐2.0 V_RHE_ steered *j*_C2+_of CuO*_x_* CPTMS to 230 mA cm^−2^ while maintaining the FE_C2+_ at 74%. At this applied potential, CuO*_x_* captured merely 6% in FE_C2+_. This significant enhancement of both FE_C2+_ and *j*_C2+_ suggests a greatly enhanced activity toward CO_2_RR after CPTMS modification. The highest CO_2_RR current density (*j_CO2RR_*) of 241 mA cm^−2^ was achieved by CuO*_x_* CPTMS at −2.0 V_RHE_ (Figure S5b). Additionally, to ensure that the CO_2_RR products were not generated from the decomposition of the carbon‐containing CPTMS, the experiment on CuO*_x_* CTPMS by using Argon (Ar) instead of CO_2_ was performed. The Ar reduction of CuO*_x_* CTPMS at −1.5 V_RHE_ resulted in FE_H2_ of 97.7%. The performance test result of Ar reduction of CuO*_x_* CTPMS at −1.5 V_RHE_ and the current‐time curve are included in Figure S6. In parallel, the CO_2_RR performance of the control samples Cl‐CuO*_x_* and CuO*_x_* PTMS was evaluated across the same potential range (Figure 2c,d). The optimum CO_2_RR performance for these catalysts was recorded at −1.5 V_RHE_ with a comparable FE_C2+_ between Cl‐CuO*_x_* and CuO*_x_* PTMS (44% and *j*_C2+_ = 56 mA cm^−2^ vs. 44% and *j*_C2+_ = 49 mA cm^−2^) at which the FE_H2_ of Cl‐CuO*_x_* was at 31% compared with 44% in CuO*_x_* PTMS (Figure S7). In terms of the total FE of CO_2_RR (FE*_CO2RR_*), Cl‐CuO*_x_* achieved 53% while CuO*_x_* PTMS recorded 51%. This phenomenon indicates that Cl‐CuO*_x_* and CuO*_x_* PTMS each contribute to enhanced CO_2_RR performance, and their concurrent presence gives rise to a synergistic effect that favors C_2+_ product formation. Nevertheless, the total FE below 100% suggested the possibility of parasitic faradaic reactions [19]. Due to the similarity in the exposed facets observed in the SR‐XRPD patterns, it is unlikely that the differences in exposed facets play a role in the variability of the CO_2_RR performance.

Due to the distinct CO_2_RR performance, the electrochemical active surface area (ECSA) was measured to identify the abundance of metal sites in CuO*_x_*, CuO*_x_* CPTMS, Cl‐CuO*_x_*, and CuO*_x_* PTMS. The electric double‐layer capacitance (C_dl_) value of CuO*_x_* and CuO*_x_* CPTMS was calculated to be 0.05 and 0.38 mF cm^−2^, corresponding to ECSA of 1.3 and 9.5 cm^2^, respectively (Figure 2e). The enhanced CO_2_RR performance may be associated with an increased local CO_2_ availability at the electrode surface induced by the higher electric double‐layer capacitance [20], which can promote CO_2_ adsorption [21]. This modified interfacial environment at the electrode–electrolyte interface may also suppress the competing HER activity on CuO*_x_* CPTMS. Additionally, the control samples Cl‐CuO*_x_* and CuO*_x_* PTMS show ECSA of 10.3 and 4.8 cm^2^, respectively. The significant decrease in ECSA observed for Cl‐free catalysts (CuO*_x_* and CuO*_x_* PTMS), highlights the essential role of Cl in preserving an active‐site‐enriched catalyst surface. To verify this postulation, Cl‐modified CuO*_x_* PTMS (Cl‐CuO*_x_* PTMS), and Cl‐modified CuO*_x_* CPTMS (Cl‐CuO*_x_* CPTMS) were synthesized. Specifically, KCl was employed as the chlorine source to potentially anchor Cl to PTMS in the creation of a close resemblance of CPTMS. The ECSA in Cl‐CuO*_x_* PTMS and Cl‐CuO*_x_* CPTMS consist of different Cl concentrations, were evaluated. As shown in Figure S8 and Table S2, increasing the Cl concentration leads to a clear enhancement in ECSA.

In addition to the ECSA, electrochemical impedance spectroscopy (EIS) was employed to analyze the charge transfer resistance [18]. The Nyquist plots (Figure S9) show two semicircles, one at high frequency, which indicates the charge transfer resistance on the electrode (R_ct1_) while the other at low frequency reflects the charge transfer between the electrode and adsorbed species (R_ct2_) at a certain applied potential [22, 23, 24]. For an easier comparison, the Nyquist data were shifted to the origin omitting the ohmic resistance. It was observed that CuO*_x_* presents the lowest R_ct1_ across all catalysts (∼ 1.3 Ω). The modification with Cl alone exhibited an increase in R_ct1_ (∼ 2.2 Ω), likely due to the incorporation of non‐conducting Cl to CuO*_x_*. In contrast, the involvement of Si as the modifier displays a relatively low R_ct1_ in the range of ∼ 1.4–1.6 Ω. Notably, although unmodified CuO*_x_* exhibits intrinsically low charge transfer resistance within the electrode, it predominantly drives the competing HER rather than contributing to CO_2_RR. While surface modification strategy inevitably introduces additional resistances, the incorporation of Si can mitigate the resistance increase induced by Cl, thereby minimizing charge‐transfer limitations while redirecting electron and proton transport toward CO_2_RR‐relevant pathways at the electrode–electrolyte interface.

Subsequently, the intrinsic activity of the catalysts as exhibited by the ECSA‐normalized C_2+_ partial current density of CuO*_x_* (Figure S10a) and CuO*_x_* CPTMS (Figure S10b) shows similar CO_2_RR activity at the applied potential above −2.0 V_RHE_. When the applied potential is decreased to −2.0 V_RHE_, CuO*_x_* CPTMS exhibited a higher ECSA‐normalized C_2+_ partial current density, suggesting a higher intrinsic activity of CuO*_x_* CPTMS compared with CuO*_x_*. Further investigation of the role of the active sites was conducted by performing linear sweep voltammetry (LSV) in Ar or CO_2_ conditions. CuO*_x_* CPTMS demonstrates CO_2_RR preference over HER across the negative applied potentials (Figure 2f). In contrast, CuO*_x_* exhibits prevalent HER over CO_2_RR (Figure S11a), as the *j* measured in an Ar environment (Ar‐CuO*_x_*) surpasses that measured in the CO_2_ environment (CO_2_‐CuO*_x_*). It is observed that the sluggish charge transfer in Cl‐CuO*_x_* due to high R_ct1_ was deprived of CO_2_RR by HER when the *j* measured in Ar environment (Ar‐Cl‐CuO*_x_*) exceeded the *j* measured in CO_2_ environment (CO_2_‐Cl‐CuO*_x_*) at the applied potential below ‐1.7 V_RHE_, despite having a relatively high ECSA value, consistent with the observed CO_2_RR performance (Figure S11b). In contrast, CuO*_x_* PTMS with a lower ECSA value shows a preference for CO_2_RR over HER at the applied potential above −1.9 V_RHE_, achieving a higher current density than that of Cl‐CuO*_x_*, likely compensated by the low R_ct1_ as compared (Figure S11c). In addition, the initial non‐zero current observed across all the LSV measured can be attributed to the double‐layer charging and self‐reduction processes at the electrode surface [25, 26]. Despite the individually enhanced ECSA in CuO*_x_* PTMS and Cl‐CuO*_x_* as compared to CuO*_x_*, a noticeable improvement in catalytic performance is observed only in the concurrent presence of Cl and Si (CuO*_x_* CPTMS), suggesting a synergistic interaction between these two modifiers. Therefore, distinct ECSA values or R_ct1_ cannot independently dissociate the changes recorded in LSV and CO_2_RR performance. Instead, the interdependency of ECSA values and R_ct1_ on the measured LSV and FE*_CO2RR_* reflected the essential interplay between Cl‐improved ECSA and Si‐contributed reduced charge transfer resistance.

**FIGURE 3:**
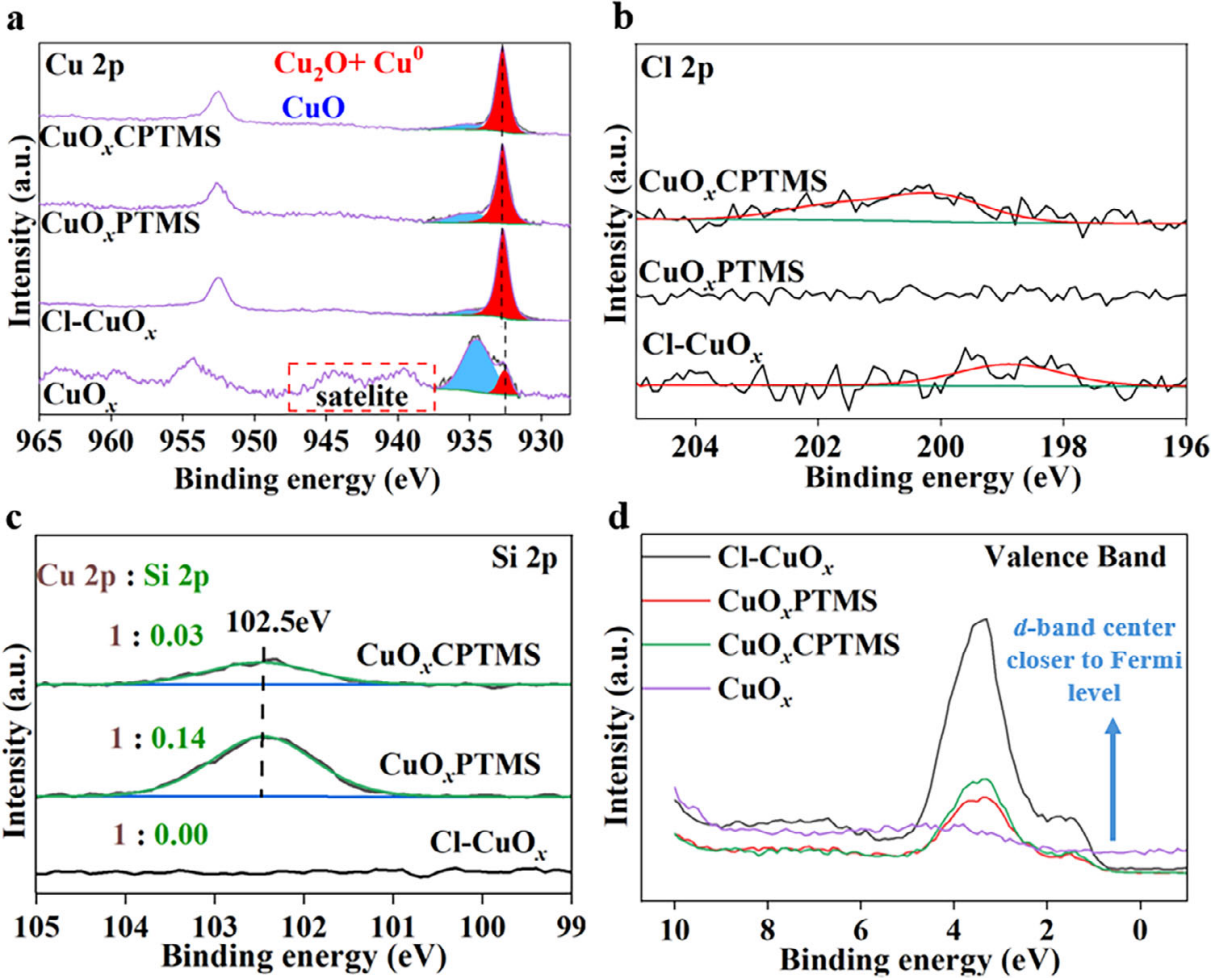
(a) Cu 2p XPS of CuO*_x_*, Cl‐CuO*_x_*, CuO*_x_* PTMS, and CuO*_x_* CPTMS, (b) Cl 2p XPS of Cl‐CuO*_x_*, CuO*_x_* PTMS, and CuO*_x_* CPTMS, (c) Si 2p XPS of Cl‐CuO*_x_*, CuO*_x_* PTMS, and CuO*_x_* CPTMS, and (d) Valence band of CuO*_x_*, Cl‐CuO*_x_*, CuO*_x_* PTMS, and CuO*_x_* CPTMS.

To explain the structure‐performance relationship, the surface chemical compositions and oxidation states of catalysts were initially explored by using X‐ray photoelectron spectroscopy (XPS) to obtain the spectra for Cu 2p, O 1s, Cl 2p, Si 2p, and valence band. The Cu 2p XPS (Figure 3a) of the samples reveals the presence of multiple Cu oxidation states across all the examined samples. The deconvoluted Cu 2p_3/2_ spectrum of CuO*_x_* shows a peak at ∼934.5 eV, corresponding to the binding energy of Cu^2+^ species, while the peak at ∼932.4 eV is attributed to a mixture of Cu^0^ and Cu^+^ species [27, 28]. Similar Cu 2p_3/2_ features are also observed for the modified catalysts Cl‐CuO*_x_*, CuO*_x_* PTMS, and CuO*_x_* CPTMS, indicating the presence of mixed Cu chemical states on these samples. However, these modified catalysts exhibit a higher proportion of Cu^0^ and Cu^+^ species, as evidenced by the larger fitted peak area relative to that of CuO*_x_*. This behavior can be attributed to the modifier layer on the surface, which suppresses oxidation of the Cu surface upon exposure to air [29, 30]. This observation is further supported by the Cu LMM Auger spectra (Figure S12a) peak at ∼ 915.6 to 916.4 eV that the peak broadening exhibited in CuO*_x_* implies a higher composition of oxidized copper compared with the sharp peak of CuO*_x_* CPTMS, Cl‐CuO*_x_*, and CuO*_x_* PTMS [28, 31].

Notably, for CuO*_x_* CPTMS, both Cu 2p_3/2_ peaks show positive binding‐energy shifts of approximately +0.5 and +0.2 eV, appearing at ∼935.0 and ∼932.6 eV, respectively. Such positive shifts indicate interfacial charge transfer, where the Cu sites act as electron donors to the CPTMS molecular modifier, resulting in increased positive charge density on the Cu species [32]. Similar positive binding‐energy shifts observed in Cl‐CuO*_x_* and CuO*_x_* PTMS, suggest a change in chemical binding environment due to the modifications by Cl or Si, which resulted in the interfacial electron delocalization around Cu sites. Overall, the modification with Cl, Si, or Si‐Cl has been shown to contribute to varying degrees of charge polarization at the Cu‐modifier interface.

Additionally, the abundance of surface hydroxyl‐associated oxygen vacancy (O_v_) was investigated by fitting the O 1s in XPS (Figure S12b) [33]. The deconvoluted O_v_ to lattice oxygen (O_lat_) peaks show an increase of O_v_:O_lat_ ratio across all the Si/Cl modified catalysts. This is most likely attributed to the changes in electrodeposition kinetics induced by the introduction of different modifiers, which promote defect formation [34]. As the CO_2_RR performance observed did not scale with the O_v_:O_lat_ ratio across all the catalysts and the earlier observed ECSA‐normalized current density of CuO*_x_* and CuO*_x_* CPTMS indicated a similar intrinsic activity at less negative applied potential, it is implied that O_v_:O_lat_ ratio is unlikely the main driver for the enhancement in C_2+_ selectivity.

The presence of Cl 2p in XPS (Figure 3b) [35, 36, 37, 38] suggested the successful modification of Cl‐CuO*_x_* and CuO*_x_* CPTMS. The presence of chlorine can also be seen in the Cl 2p of Cl‐CuO*_x_* PTMS and Cl‐CuO*_x_* CPTMS (Figure S12c). Furthermore, the peaks shown in Si 2p at 102.5 eV indicated the presence of Si─O species contributed by the Si in silane (Figure 3c). The successful modification was demonstrated by Cl‐CuO*_x_* PTMS and Cl‐CuO*_x_* CPTMS (Figure S12d) via XPS. Additionally, nano‐IR s‐SNOM technique (Figure S13) was applied to confirm the observation in Si 2p. Besides Cu_2_O at ∼ 645 cm^−1^ [39] the absorption band at ∼1100–1000 cm^−1^ attributed to the Si─O species [40, 41] was detected on CuO*_x_* CPTMS, supporting the findings from XPS and EDS. In addition, the valence band was collected (Figure 3d and Figure S14) to investigate the *d‐*band centers of the catalysts, as variations in the *d‐*band center are known to influence the adsorption strength between metal sites and adsorbates [42]. The shifted *d*‐band center closer to the Fermi level as calculated (Table S3) [43, 44] from the valence band is observed across all modified catalysts, due to the electron redistribution contributed by electron‐withdrawing Cl [45] and σ‐donating Si [46]. Specifically, Cl‐CuO*_x_*, Cl‐CuO*_x_* PTMS, and Cl‐CuO*_x_* CPTMS exhibited shifted *d*‐band centers closer to the Fermi level than the catalysts synthesized without KCl in the electrodeposition bath. The shift of the *d‐*band center closer to the Fermi level suggests more energetically favorable π‐ backdonation and may cause less filling of the antibonding states, hence stronger binding strength of CO_2_, compared with Si‐modified catalysts [47, 48], provided that the electron density among the modified catalysts is comparable. As the XPS valence band reflects the density of states (DOS) near the Fermi level [49, 50], the results indicated that the change in the *d*‐band center is highly dependent on the presence of Cl in the modifiers.

Nonetheless, the presence of lattice‐bound Cl in Cl‐CuO*_x_* was not commensurate with the CO_2_RR performance of CuO*_x_* CPTMS, despite having a *d*‐band center closer to the Fermi level nor was CuO*_x_* PTMS with a *d*‐band center further from the Fermi level than Cl‐CuO*_x_* exhibited impeded CO_2_RR performance relative to Cl‐CuO*_x_*. Therefore, it is hypothesized that Cl and Si enhance CO_2_RR via different mechanisms i.e. Cl‐driven strong π‐ backdonation for CO_2_ activation and the delocalized Cu *d*‐electrons in CuO*_x_* PTMS reduce the free energy barriers for *COOH intermediate formation [51, 52]. Consequently, CuO*_x_* CPTMS which consists of both Si and Cl manifested enhanced CO_2_RR performance, particularly toward C_2+_ product formation. Considering the aforementioned investigations, it is posited that CPTMS has benefited from the combined roles of Si and Cl in enhancing the ECSA, lowering R_ct1_ despite having similar Cu species and exposed facets. Besides, the CO_2_RR performances of CuO*_x_* PTMS with the highest O_v_:O_lat_ ratio and CuO*_x_* with the lowest O_v_:O_lat_ ratio are not comparable to the CO_2_RR performance of CuO*_x_* CPTMS with a moderate O_v_:O_lat_ ratio. Therefore, O_v_:O_lat_ ratio is unlikely to be definitive of CO_2_RR performance.

On the other hand, as compared to CuO*_x_*, the improved performance from Cl‐CuO*_x_* could potentially arise from the modification of the catalyst with Cl. However, it is not clear if the enhancement was due to the weakly bound Cl atom to the Cu surface or the lattice‐bound Cl incorporated into the catalysts. Therefore, the interaction between Cl and the catalysts on CO_2_RR performance was further investigated by using the catholyte with 0.5 m KCl as an additive to the 1 m KOH. The examined CO_2_RR showed an overall increase in the total current density (*j*_total_) due to the increase in ion participation from a more concentrated catholyte (Figure S15). With regard to CO_2_RR performance, the reduced ionic resistance and enhanced kinetics from the concentrated catholyte have advanced the competing HER in CuO*_x_*. In contrast, comparable selectivity on the CuO*_x_* CPTMS catalyst was observed (FE_C2+_ of 77%), but at a higher *j*_C2+_ at 171 mA cm^−2^. Additionally, Cl‐CuO*_x_* showed a slightly boosted FE_C2+_ at 52%, likely due to the promoted electron transfer. In addition, the hampered CO_2_RR performance in CuO*_x_* PTMS succumbed to the competing HER was recorded (Figure S15g). As the selectivity toward CO_2_RR was not observed to be shifted when 0.5 m KCl was introduced as the additive to the catholyte of 1 m KOH, it can therefore be concluded that Cl participated in the CO_2_RR as lattice‐bound Cl to the catalysts.

### 2.2 Mechanistic Insights Into CO_2_RR Pathways and Intermediates

To validate our hypothesis on the individual roles of Si and Cl in promoting C_2+_ product formation, in‐situ synchrotron‐based Fourier transform infrared (SR‐FTIR) microspectroscopy was employed to probe the CO_2_RR intermediates formed on CuO*_x_* and CuO*_x_* CPTMS surfaces under applied cathodic potentials. Specifically, the collected spectra were analyzed to identify distinct spectral features that differentiate the catalytic behaviors of the catalysts, providing insights into the origin of the enhanced C_2+_ production.

**FIGURE 4:**
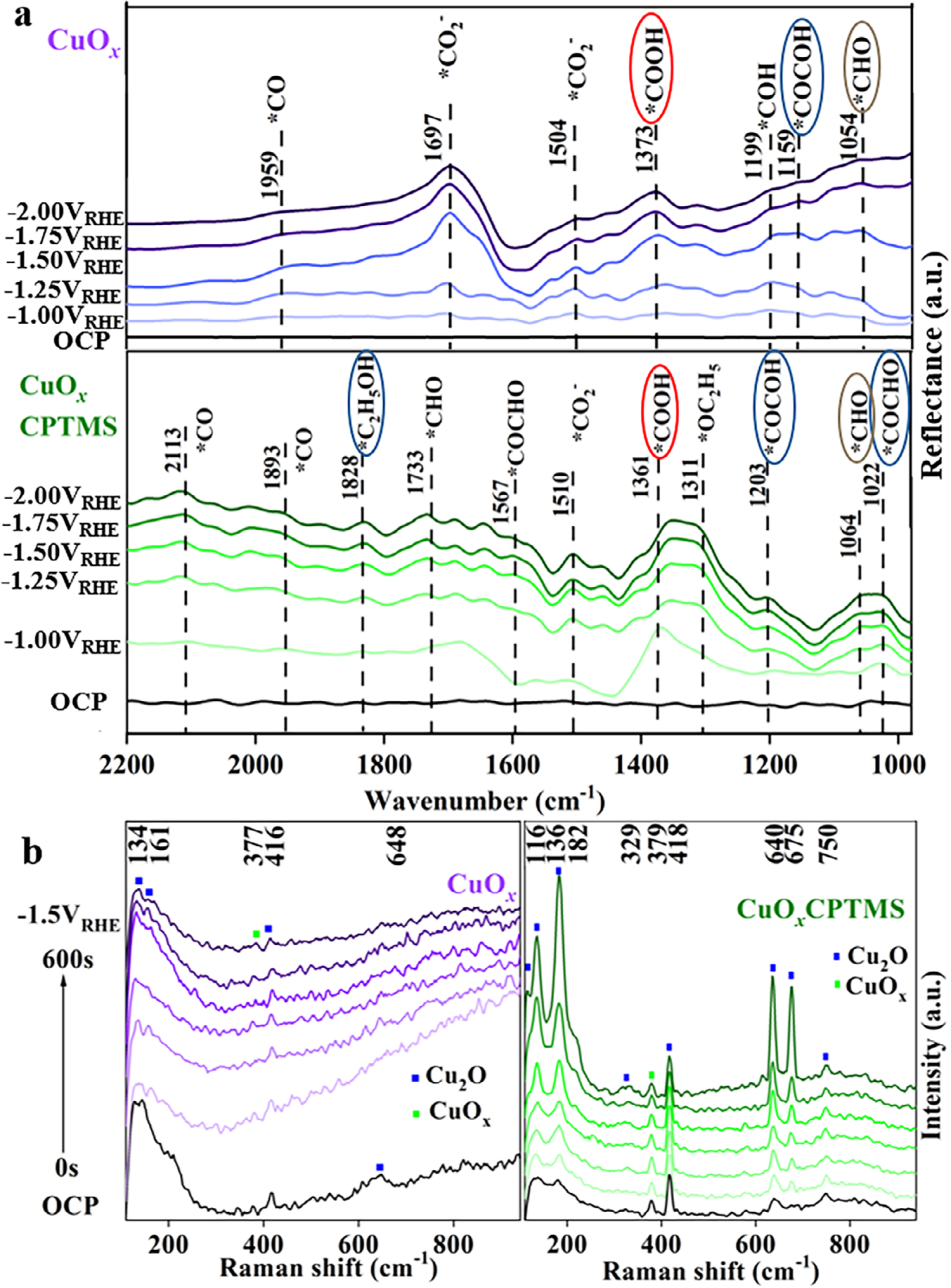
(a) Insitu SR‐FTIR and (b) in‐situ Raman spectra recorded during CO_2_RR on CuO*_x_* and CuO*_x_* CPTMS surfaces.

Among the commonly observed CO_2_RR intermediates (Table S4), the most prominent contrast between the examined catalysts appears to be the vibrational modes at 1373 and 1361 cm^−1^ with positively increasing intensity (Figure 4a), indicative of progressive formation of *COOH species (red circle) [53]. In addition, the notable difference of the absorption bands was observed at 1064 and 1054 cm^−1^, attributed to *CHO intermediates (brown circle) [54, 55]. Distinct vibrational modes associated with C_2+_ products are also observed (blue circle), including *COCHO (∼1022 cm^−1^), *COCOH (1203–1159 cm^−1^), and *C_2_H_5_OH (∼1828 cm^−1^) [56, 57, 58].

The key distinction between CuO*_x_* and CuO*_x_* CPTMS lies in the onset potential for *COOH formation. On CuO*_x_*, *COOH is detected only at more negative applied potentials, whereas *COOH accumulation on CuO*_x_* CPTMS is observed immediately upon application of a cathodic potential (red arrows). This behavior indicates a facilitated CO_2_ adsorption and activation process in the presence of Cl [7]. In particular, *COCHO intermediates are also detected at 1022 cm^−1^ at the onset of cathodic potentials, which is in marked contrast to their absence on CuO*_x_*. The rapid emergence of *COCHO, followed by *COCOH, and *C_2_H_5_OH in CuO*_x_* CPTMS, suggests an accelerated C─C coupling on CuO*_x_* CPTMS, which is attributed primarily to the incorporation of Si‐reduced R_ct1_.

To further investigate the individual contributions of Cl and Si in the CO_2_RR, control in‐situ SR‐FTIR experiments were conducted on Cl‐CuO*_x_* and CuO*_x_* PTMS. Interestingly, *COOH formation is detected immediately on Cl‐CuO*_x_* upon applying cathodic potential, whereas no discernible *COOH signal is observed on CuO*_x_* PTMS throughout the experiment (Figure S16). This phenomenon hence supports that the role of Cl in enhancing CO_2_ adsorption and activation when the *d‐*band center is closer to the Fermi level [48]. consistent with strengthened *COOH stabilization on the catalyst surface. In contrast, CuOxPTMS exhibits progressively increasing intensities of *COH and *CHO intermediates, indicating that Si promotes protonation of *CO‐derived species. However, despite the abundance of *COH and *CHO, CuO_x_PTMS shows no pronounced accumulation of C_2+_ intermediates, suggesting that *C─C coupling remains limited by insufficient CO availability, originating from the lack of *COOH formation.

As the precursor to the formation of C_2_H_4_, *COCHO intermediates appear earlier in Cl‐CuO*_x_* than on CuO*_x_*, likely benefiting from the ready availability of *CO derived from *COOH as soon as cathodic potential was applied or due to a mixed Cu species participating during the CO_2_RR [16]. However, the *COCHO signal diminishes at more negative potentials, suggesting limited stabilization of downstream C─C coupling intermediates in the absence of Si. Overall, CuO*_x_* CPTMS uniquely demonstrates sustained accumulation of C_2+_ intermediates, arising from the synergistic combination of Cl‐promoted *COOH formation and Si‐facilitated protonation of *CO to *COH and *CHO. Besides, the concurrent detection of *COCHO and *COCOH indicates the co‐existence of multiple C─C coupling pathways in CuO*_x_* CPTMS, proceeding via either 2CO_2_→ 2*COOH→ 2*CO→ *CO+*CHO→ *OCCHO or 2CO_2_→ 2*COOH→ 2*CO→ *CO+*COH→ *OCCOH, with the former pathway appearing kinetically favored due to its earlier onset. From a mechanistic perspective, the synergistic effect between Si and Cl enables the sequential formation of key intermediates required for C_2+_ product generation, which is unattainable on Cl‐CuO*_x_* or CuO*_x_* PTMS alone.

Subsequently, in‐situ Raman spectroscopy was employed to assess whether variations in Cu oxidation states contribute to the observed selectivity differences in CuO*_x_* and CuO*_x_* CPTMS (Figure 4b). At open circuit potential (OCP), vibrational bands in the ranges of 134–329 cm^−1^ and 416–750 cm^−1^ are assigned to Cu_2_O, while the band at 377–379 cm^−1^ corresponds to an electron‐deficient Cu (Cu^δ+^) [59], attributed to surface CuO_x_ species [60]. Upon applying a cathodic potential of −1.5 V_RHE,_ CuO*_x_* largely retains its oxidized Cu species, with partial reduction of Cu_2_O evidenced by the attenuation of the band at 648 cm^−1^. The intermittent appearance of Cu_2_O‐related features at 161–134 cm^−1^ suggests the reduction of Cu (I) species at this vibration was compensated by the stable reduction of Cu (II) species [60]. The absence of hydroxide‐related Raman bands (at ∼460 and ∼520 cm^−1^) and the lack of hydroxide signatures in XRD indicate that Cu (II) species predominantly originate from CuO, consistent with XPS results showing a high Cu^2+^ fraction of 85% (Figure 3a).

In contrast, CuO*_x_* CPTMS exhibits the emergence and increased intensities of Cu_2_O‐associated bands at 116, 329, and 675 cm^−1^ under cathodic potential, together with the enhanced features at 136, 182, 640, and 675 cm^−1^. These results indicate a more pronounced reduction of Cu (II) species during CO_2_RR or a redistribution of electron density induced by Si and Cl incorporation. Similar oxidized Cu species were observed in Cl‐CuO*_x_* and CuO*_x_* PTMS during CO_2_RR (Figure S17). Despite a lower fraction of CuO species as probed by XPS Cu 2p (Figure 3a) as well as from the integrated area of CuO (−113) species from ex‐situ SR‐XRPD (Figure 1a and Table S5), the oxidized copper species was stabilized in CuO*_x_* CPTMS throughout the reaction likely due to the electrons withdrawal effect from Cl and the strengthened Cu─O─Si bond [7, 61, 62, 63, 64]. *Ex‐situ* Raman spectroscopy was employed in investigating the copper species on CuO*_x_* and CuO*_x_* CPTMS (Figure S18). The results confirmed the origin of the emergence of Cu_2_O during in‐situ Raman spectroscopy from the detected CuO (Figure S18), substantiating the observation of CuO species in SR‐XRPD and XPS. As Cu^0^ species is silent in Raman spectroscopy, the evolution of Cu^0^ species accompanied the variation in Cu^+^ species in CuO*_x_* CPTMS was further probed via in‐situ SR‐XRPD (Figure S19). The peaks associated with Cu (111) at 2θ = 16.3° (0.5904 Å) and Cu (002) at 2θ = 18.8° (0.5904 Å) were detected throughout CO_2_RR. Upon integration, the area of Cu (111) and Cu (002) was increased by 1.02 and 1.08 times, respectively, at 600^th^ second compared with the areas at 0^th^ second (Table S5), suggesting the comparatively stabilized Cu^+^ species in CuO*_x_* CPTMS with minimal reduction of Cu^+^ species. On the other hand, the progressive appearance of Cu_2_O (113) peak at 2θ = 26.5° (0.5904 Å) was detected, echoing the observation on the participation of oxidized copper species in CO_2_RR during in‐situ Raman experiment. In addition, *ex‐situ* XRD measurements after CO_2_RR confirm that the bulk crystallinity of all catalysts (i.e. CuO*_x_*, CuO*_x_* CPTMS, Cl‐CuO*_x_*, and Cl‐CuO*_x_* PTMS) was preserved, with partial re‐oxidation to Cu(OH)_2_ and CuO observed post‐reaction (Figure S20). Furthermore, SEM analysis reveals comparable particle aggregation across all the samples (Figure S21).

To validate the synergistic role of Si and Cl, additional studies were conducted on Cl‐CuO*_x_* PTMS. Electrochemical CO_2_RR performance in 1 m KOH closely resembles that of CuO*_x_* CPTMS (Figure S22a,b), while the addition of 0.5 m KCl to the electrolyte does not yield similar enhancements (Figure S22c,d), indicating that surface‐bound Cl rather than electrolyte Cl^−^ is responsible for the observed effects. In terms of the CO_2_RR mechanism probed by in‐situ SR‐FTIR microspectroscopy, spectra of Cl‐CuO*_x_* PTMS reveal strong *COOH signals (Figure S22e), consistent with its higher Cl content in Cl‐CuO*_x_* PTMS compared with CuO*_x_* CPTMS (5 vs. 2.7 mm). The concurrent detection of *COCHO, together with a downward feature at *CHO despite abundant *COOH, suggests rapid consumption of *CHO toward C‐C coupling and C_2_H_4_ formation. This interpretation aligns with the higher electrochemically active surface area (ECSA) and lower charge‐transfer resistance R_ct1_ measured for this catalyst (Figures S8 and S9).

Overall, these results demonstrate that the cooperative interaction between Si and Cl enables the sequential formation of key CO_2_RR intermediates (i.e. *COOH, *CO, *COH/*CHO, and C─C coupling species), culminating in enhanced C_2+_ product formation, a pathway that is not accessible when either component is present alone.

### 2.3 Theoretical Studies

**FIGURE 5:**
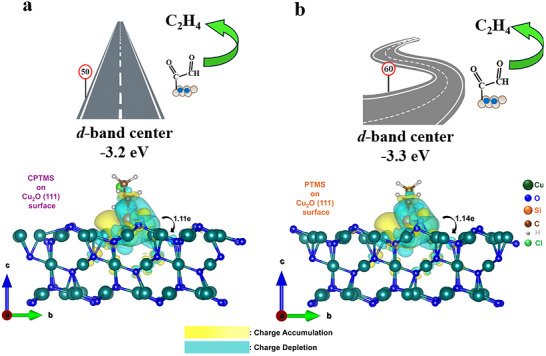
Charge transfer and *d‐*band center of (a) Cu_2_O (111)‐CPTMS and (b) Cu_2_O (111)‐PTMS.

The bonding configurations between silane molecules to the surface of CuO*_x_* were analyzed by density functional theory (DFT) calculation. To account for incompletely reduced Cu species as active sites, Cu_2_O (111) was selected as the basis for the computational models (Figure S23a). Accordingly, DFT calculations were performed to model pristine CuO*_x_* without modification, with PTMS (Figure S23bi) and with CPTMS (Figure S23bii) modifications, respectively. Namely, Cu_2_O (111), Cu_2_O (111)‐PTMS and Cu_2_O (111)‐CPTMS were modeled. The results illustrate that silane molecules interacted with Cu catalyst via Si─O bonds in PTMS and CPTMS (Figure S23c,d), suggesting that PTMS and CPTMS were successfully hydrolyzed to highly active silanol for the binding to Cu surface during electrodeposition [65]. Upon functionalization with PTMS and CPTMS, the *d*‐band center of Cu_2_O (111)‐CPTMS is calculated to be closer to the Fermi‐level at −3.2 eV, compared with Cu_2_O (111)‐PTMS at −3.3 eV, echoing the calculated *d‐*band center from the valence band acquired in XPS ((Figure 3d). While the magnitude of the shift in DFT is smaller than that observed experimentally (0.24 eV; Table S3), this difference may arise from the simplified model used in the DFT calculations i.e. limited surface coverage of CPTMS and PTMS on the Cu_2_O (111) slab, respectively. To investigate the charge transfer imparted by PTMS and CPTMS, we performed Bader charge analysis on Cu_2_O (111)‐PTMS and Cu_2_O (111)‐CPTMS. The analysis reveals a higher charge transfer of Cu_2_O (111)‐PTMS, compared with Cu_2_O (111)‐CPTMS (Figure 5), consistent with the higher R_ct1_ measured in Cl‐CuO*_x_* i.e. the charge transferred is enhanced by Si‐involvement. Overall, Cu_2_O (111)‐CPTMS and Cu_2_O (111)‐PTMS demonstrate the transfer of electrons to the Cu sites, implying a stronger back‐bonding to the adsorbates [66], consistent with the suggested energetically favorable π‐backdonation in silane‐modified catalysts which leads to enhanced C_2+_ product formation. In parallel, less filling of the antibonding states resulted from a shifted *d‐*band center closer to the Fermi level in CuO*_x_* CPTMS, causing more electrons to transfer from the Cu sites to the antibonding orbitals of CO_2_ molecules hence enhancing CO_2_ activation [67]. Despite a *d‐*band center further from the Fermi level, the hypothesized reduced intermediate formation energy resulted from the delocalization [51] of *d‐*electron in Cu_2_O (111)‐PTMS is further benefited from the moderate charge transfer from PTMS to the Cu metal sites as compared with CuO*_x_*. The embodiment of CuO*_x_* CPTMS with the Si‐driven higher charge transfer kinetics, electronic delocalization, and Cl‐driven π‐backdonation distinguishes the C_2+_ products formation capability from Cl‐CuO*_x_* and CuO*_x_* PTMS, which consist of only a single enhancement descriptor.

## 3 Conclusion

In summary, this work establishes a molecularly integrated halide‐silane strategy to cooperatively regulate the multi‐step CO_2_ reduction to C_2+_ products on Cu based catalysts. By rationally designing chlorine‐functionalized silane (i.e. CPTMS) as a single‐molecule modifier, we achieve spatially coupled interfacial control that unifies CO_2_ activation, charge transfer regulation, and C─C coupling within one scaffold. In particular, CuO_x_CPTMS achieves a 5‐fold enhancement in FE_C2+_ compared with CuO_x_ (75% vs. 15%) at −1.5 V_RHE_. Mechanistic investigations reveal a sequential and cooperative role of the two functionalities. Cl enables an enhanced CO_2_ activation through a stronger π‐ backdonation of Cu active sites closer to the Fermi level, while silane anchoring regulates charge transfer, stabilizes protonated *CO‐derived intermediates, and lowers the kinetic barrier for C–C dimerization, evidenced by instant accumulation of protonated intermediates in SR‐FTIR. Together, these effects enable stepwise optimization of the CO_2_RR pathway from CO_2_ activation, *CO formation to *CO protonation and C─C coupling for C_2+_ products. Concurrently, the competing HER has also been suppressed through interfacial microenvironment modulation. This study establishes a rational catalyst design paradigm via a molecular design framework, guided by element‐specific electronic, and interfacial functionalities for advancing selective C_2+_ product formation in CO_2_RR.

## 4 Experimental Section

### 4.1 Chemicals

Copper (II) sulfate pentahydrate (CuSO_4_·5H_2_O; 90%) potassium chloride (KCl; 99%) and potassium hydroxide (KOH; 90%) were purchased from Chem‐Supply. A certificate of analysis of 90% KOH is provided in Figure S24. 99.98% KOH (ThermoFisher Scientific) was used to validate the CO_2_RR performance test on CuO*_x_* CPTMS performed with 90% KOH to ensure the validity of the product distributions presented in Figure 1b (Figure S25). As the major impurity in the 90% KOH is water, while the other known impurities are believed to be negligible in affecting the electrochemical performance, the electrochemical tests in this work were conducted using 90% KOH, to be more cost‐efficient. Pluronic F‐127 and 3‐Chloropropyltrimethoxysilane (CPTMS; 98%) were purchased from Sigma–Aldrich, while propyltrimethoxysilane (PTMS; 98%) was purchased from TCI Chemicals. Gas diffusion electrode and iridium oxide (IrO_2_) anode were purchased from Dioxide Materials. Ag/AgCl reference electrode, graphite counter electrode, and anion exchange membrane (AEM) were purchased from Gauss Union. All solutions were prepared using Milli‐Q water (18.2 MΩ·cm).

### 4.2 Synthesis

5 mm of CuSO_4_·5H_2_O electrodeposition bath is prepared in a 120 mL beaker added with 1 g of pluronic F‐127 as the base solution. The adjusted electrodeposition procedure from Arán‐Ais et al. was adopted [68]. The copper particles of mixed oxidation states (CuO*_x_*) were grown in CuSO_4_·5H_2_O solution by electrochemical cycling between −0.2 and −0.5 V (vs. Ag/AgCl) at a sweep rate of 5 mV s^−1^. 0.5 mL of CPTMS is added to the base solution for the electrodeposition of CuO*_x_* CPTMS while 0.48 mL of PTMS is added to the base solution for the electrodeposition of CuO*_x_* PTMS. 5 mm of KCl is employed as the source of Cl in the base solution for the electrodeposition of Cl‐CuO*_x_*, Cl‐CuO*_x_* PTMS, and Cl‐CuO*_x_* CPTMS. The electrodeposited electrode (working electrode; WE) was rinsed with a copious amount of Mili‐Q water and left air‐dry overnight.

### 4.3 *Ex‐Situ* Characterizations

FEI Nova field‐emission scanning electron microscopy (NanoSEM 230) was employed to examine the morphologies of the catalysts before and post reaction. XRD was performed using an Aeris Benchtop XRD system. Thermo ESCALAB250Xi spectrometer with a 500 µm spot size was used for XPS acquisition; the binding energy was corrected by the C 1s peak of 284.8 eV. CasaXPS software was used for XPS fittings. JEM‐F200 was employed in acquiring the TEM images. EDS mapping on CuO_x_CPTMS was performed using a JEOL JSM‐7001F. *Ex‐situ* SR‐XPRD experiments were performed at λ = 0.7748 Å (Figure 1a).

### 4.4 in‐situ Characterization

In‐situ synchrotron radiation Fourier transform infrared (SR‐FTIR) microspectroscopy was acquired on the Infrared Microspectroscopy (IRM) beamline at the Australian Synchrotron (Clayton, Victoria) using a custom designed in‐situ FTIR cell with a 0.5‐mm‐thick ZnSe top window (Figure S26). The IRM beamline was equipped with a Bruker Vertex 80v spectrometer coupled to a Hyperion 3000 FTIR microscope and a liquid nitrogen‐cooled narrow‐band mercury cadmium telluride (MCT) detector (Bruker Optik GmbH, Ettlingen, Germany). All SR‐FTIR spectra were collected in reflectance mode using a 20× objective (N.A. = 0.60) within a specified spectral range of 3800–750 cm^−1^ and 4 cm^−1^ spectral resolution. Blackman‐Harris 3‐Term apodization, Mertz phase correction, and zero‐filling factor of two were set as default acquisition parameters using OPUS 8 software suite (Bruker Optik GmbH, Ettlingen, Germany). In‐situ Raman spectra were performed using Renishaw inVia Reflex with a 514 nm laser diode as the excitation source, coupled with the same cell used for in‐situ SR‐FTIR technique (Figure S26). In‐situ SR‐XPRD experiment was performed at λ = 0.5904 Å (Figure S19).

### 4.5 Electrocatalytic Performance Tests

The CO_2_ reduction reaction (CO_2_RR) performance of the as‐synthesized catalysts was evaluated in a three‐electrode flow cell. An Ag/AgCl electrode (stored in saturated KCl, Gauss Union) was used as the reference electrode in the catholyte chamber, while a commercial Iridium oxide (IrO_2_) served as the counter electrode (Dioxide Materials). A pretreated anion exchange membrane (AEM) from Gauss Union was employed to separate the cathode and anode compartments. Electrochemical measurements were conducted using an Autolab M204 and Booster 10 A electrochemical workstation (Metrohm Autolab). All the electrochemical tests were conducted without iR compensation. All electrical measurements were performed under identical experimental conditions, ensuring comparable Ohmic contributions across samples. The uncompensated Ohmic resistance determined by EIS in the flow cell differs by ∼ 0.1 Ω between CuO*_x_* and CuO*_x_* CPTMS (Figure S27). These results indicate that Ohmic losses are effectively consistent across CuO*_x_* and CuO*_x_* CPTMS and do not influence the comparative trends from their individual performance. Therefore, while iR correction was not explicitly applied, the relative performance trends reported here remain valid. To assess the electrochemical active surface area (ECSA), cyclic voltammetry (CV) curves were recorded for the catalysts in the non‐faradaic region at different scan rates (20, 40, 60, 80, and 100 mV s^−1^ for CuO*_x_*, Cl‐CuO*_x_*, CuO*_x_* PTMS, and Cl‐CuO*_x_* CPTMS and 100, 200, 300, 400, and 500 mV s^−1^ for CuO*_x_* CPTMS), respectively (Figure S28). The corresponding capacitive currents were analyzed and plotted as a function of the scan rate (Figure 3a). To calculate the ECSA, the specific capacitance was referenced to an atomically smooth planar surface [69] with a real surface area of 1.0 cm^2^, where the specific capacitance (C_s_) in alkaline media typically falls within the range of 20–60 µF cm^−2^. By using the midpoint value of 40 µF cm^−2^ for C_s_ and the equation of ECSA = C_dl_ / C_s_, the ECSA values were calculated. LSV curves were recorded at the scan rate of 10 mV s^−1^. The gas products were analyzed by gas chromatography (GC‐2010, Shimadzu) and the liquid products were analyzed by nuclear magnetic resonance (Bruker Avance III 400 MHz NMR). The FE calculation is computed according to the following equation:

$$\mathrm{FE}\;\left(\%\right)=\frac{z\times n\times F}{Q}\times 100$$

where, *z* represents the number of electrons required to produce a specific product; *n* represents the molar amount of the product calculated based on GC or NMR quantification; F is the Faraday constant (96 485 C mol^−1^); *Q* represents the total charge consumed during the CO_2_RR process.

## Author Contributions

Y.Y.C performed conceptualization, methodology, investigation, formal analysis, writing‐ original draft preparation. M.Z., S.Z., and Z.H. performed supervision, conceptualization, resources, methodology, investigation, formal analysis, validation, writing‐ original draft preparation, reviewing & editing. L.J. performed conceptualization, methodology, supervision and investigation. S.S. and P.V.K. performed methodology, investigation, formal analysis. P.R. supported IRM beamtime, writing‐ reviewing & editing. J.V. supported in‐situ SR‐FTIR data acquisition, formal analysis, writing‐ reviewing & editing. A.C. performed nano‐IR data acquisition, formal analysis. Y.S. performed TEM data acquisition. L.J., Z.M., and X.L. supported with resources. R.D. supported with resources, funding acquisition, writing‐ reviewing & editing. R.A. performed supervision, conceptualization, resources, methodology, funding acquisition, investigation, formal analysis, validation, writing‐ original draft preparation, reviewing & editing.

## Funding

Open access publishing facilitated by University of New South Wales, as part of the Wiley ‐ University of New South Wales agreement via the Council of Australasian University Librarians.

## Conflicts of Interest

The authors declare no conflicts of interest.

## Supporting information




**Supporting File**: advs76741‐sup‐0001‐SuppMat.docx.

## Data Availability

The data that support the findings of this study are available from the corresponding author upon reasonable request.
