# Supplementary material for: Chlorine‐Functionalized Silane‐Modified Copper Electrocatalyst for Enhanced CO2 Reduction to Multi‐Carbon Products
Source: Adv Sci (Weinh). 2026 Jul 24:e76741. Online ahead of print. doi: 10.1002/advs.76741 (PMC13398128; doi:10.1002/advs.76741)
Supplement: Supplementary file 1 — Supporting File: advs76741‐sup‐0001‐SuppMat.docx. [file ADVS-9999-e76741-s001.docx]

**Supporting Information**

**Chlorine-Functionalized Silane Modified Copper Electrocatalyst for Enhanced CO_2_ Reduction to Multi-carbon Products**

*Ying Ying Ch’ng, Sankhadip Saha, Ming Zhang^*^, Shujie Zhou^*^, Lixue Jiang, Putri Ramadhany, Jitraporn Vongsvivut, Adrian Cernescu, Priyank Vijaya Kumar, Yihao Shan, Zhipeng Ma, Xiaoxuan Luo, Rahman Daiyan, Zhaojun Han^*^, Rose Amal*

**1. Electrochemical Tests**

Flow cell was employed in conducting the electrochemical CO_2_RR with catalyst synthesized *via* electrodeposition method (0.1 mg cm^-2^) on a hydrophobic gas diffusion electrode (GDE) as the working electrode. Ag/AgCl electrode was used as a reference electrode while Iridium oxide (IrO_2_) employed as the oxygen evolution reaction (OER) anode in this 3-electrode flow cell.

1 M KOH is used as both catholyte and anolyte. 30 mL of catholyte and anolyte are circulated in the flow-cell at 10 mL/min controlled by peristaltic pumps (Kamoer DIP 1500). CO_2_ is fed at 100 mL/min to the gas chamber regulated by the flowmeter (Agilent ADM Flow Meter). Chronoamperometry method is used for CO_2_RR performance evaluation whereby each test is run for 600 s. Each error bar was obtained by two independent electrochemical measurements. The applied potential with reference to the reversible hydrogen electrode (RHE) was converted using the following equation^[1]^:

$E_{RHE} \left( V \right)= E_{Ag/AgCl} \left( V \right)+0.197\left( V \right)+0.0591\times pH$ [1]

To prepare the NMR^[2]^ samples, 100 μL of D_2_O is mixed with 500 μL of liquid sample from CO_2_RR. The Faradaic efficiency (FE) of the liquid products was computed by using the following equation.

| $\mathrm{FE}_{\mathrm{liquid}}\text{ }\text{(\%) =}\frac{\text{z × }\text{F}\text{ × }\text{C}_{\text{Product}}\text{ × }\text{V}}{\text{Q}}\times100\%$ |  |
| --- | --- |

where *z* is the number of electrons transferred, F is the Faraday constant (96485.33 C mol^-1^), *V* is the volume of catholyte solution (L), *C*_Product_ is the concentration of product (M), and *Q* is the total charge passed (C).

For every run, 100 μL of gas product is collected and analyzed by gas chromatography. Gas products were quantified by using the following equation:

| $\mathrm{FE}_{\mathrm{gas}}\text{ (\%) =}\frac{\text{z × F × x × q}}{j\times A\times V_{g}}\times100\%$ |  |
| --- | --- |

Where *x* is the volume of the gas produced, *q* is the gas flowrate at the flow cell outlet (mL min^-1^), *j* is the current density (A cm^-2^), and *V_g_* is the unit molar volume of gas at room temperature and pressure (24.0 L mol^-1^).

**2. Computational Method**

Electronic structure calculations were performed using Density Functional Theory (DFT), as implemented in the plane-wave VASP^[3,4]^ code. Core-valence electron interactions were treated using the Projector Augmented Wave^[5]^ (PAW) method, and the Perdew-Burke-Ernzerhof^[6]^ (PBE) exchange-correlation functional was employed to solve the Kohn-Sham equations^[7]^. A Г-centred k-point mesh of 1x1x1 was used for the surface calculations, and the plane-wave was expanded with a kinetic energy cutoff of 500eV. The bulk Cu_2_O structure was obtained from the Materials Project^[8]^ database and fully relaxed using appropriate k-point sampling. The Cu_2_O (111) surface was generated by cleaving the optimized bulk structure using the Atomistic Simulation Environment^[9]^ (ASE) package. All the surface simulations were carried out using a 3x3x1 supercell of the Cu_2_O-(111) slab. To address the excessive delocalisation of the 4d electrons in Cu, the Dudarev *et al.*^[10]^ formalism with an effective U value of 7eV^[11]^ was employed. To simulate both surface- and bulk-like regions in the slab, the central layer was fixed at its optimised positions, whereas the top and bottom layers were allowed to relax. The initial geometries of the molecules: polymethoxysilane (PTMS, PubChem CID: 61254) and 3-Chloropropyltrimethoxysilane (CPTMS, PubChem CID: 62449), were taken from the PubChem^[12]^ database. Long-range dispersion and van der Waals interactions were accounted for using Grimme’s DFT-D3^[13]^, ensuring an accurate description of the intermolecular interactions and molecule-surface binding.

The adsorption energy of the molecules on the substrate was calculated using the equation:

$E_{ads}^{Molecule+Surface}= E_{Total}^{Molecule+Surface}-(E_{Total}^{Molecule}+E_{Total}^{Surface})$ [2]

Where $E_{ads}^{Molecule+Surface}$ is the adsorption energy of the cluster on the surface, $E_{Total}^{Molecule+Surface}$ is the total energy of the combined molecule/surface system, $E_{Total}^{Molecule}$ and $E_{Total}^{Surface}$ are the total energies of the individual molecules and the surface slab, relaxed to their optimized geometries, respectively.

Interfacial charge transfer between the surface and the adsorbed molecule was analysed using Bader^[14]^ charge analysis and corroborated by charge density difference (CDD). The mathematical representation of CDD is as follows:

CDD = ρ_surface+molecule_ – (ρ_surface_ + ρ_molecule_) [3]

Where ρ_surface+molecule_ represents the charge density for the combined molecule-surface system, ρ_surface_ corresponds to the charge density of the isolated surface, and ρ_molecule_ is the charge density of the isolated molecule.

To investigate the CO_2_ reaction pathway and conversion mechanism, three model systems were examined: the pristine Cu_2_O (111) surface, the PTMS-Cu_2_O (111) surface, and the CPTMS-Cu_2_O (111) surface.

**2. Supplementary Figures**


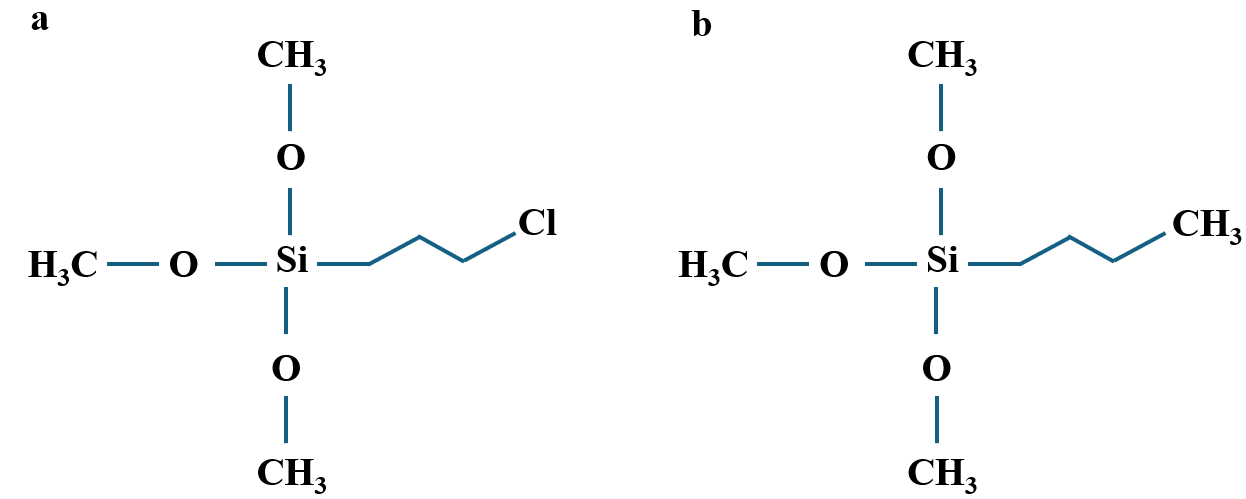


**Figure S1.** Chemical formulae of (a) 3-chloropropyltrimethoxysilane; CPTMS, and (b) propyltrimethoxysilane; PTMS


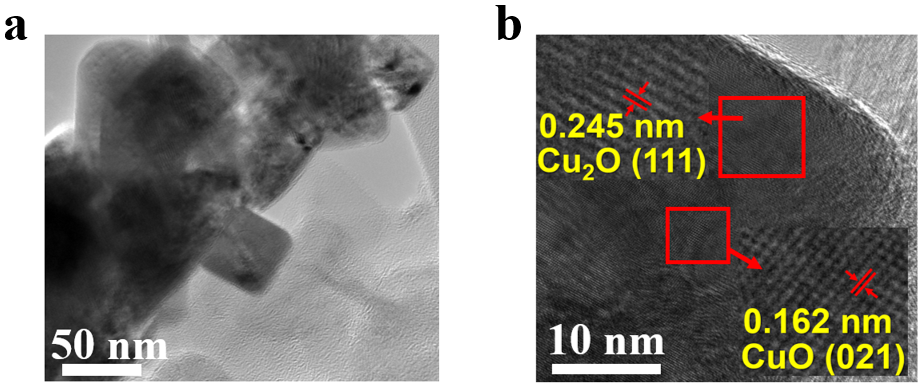


**Figure S2.** (a) TEM image of CuO*_x_* at 50nm scale, and (b) TEM image of CuO*_x_* at 10nm scale with lattice fringes. Inset shows the lattice spacing* of the probed area.


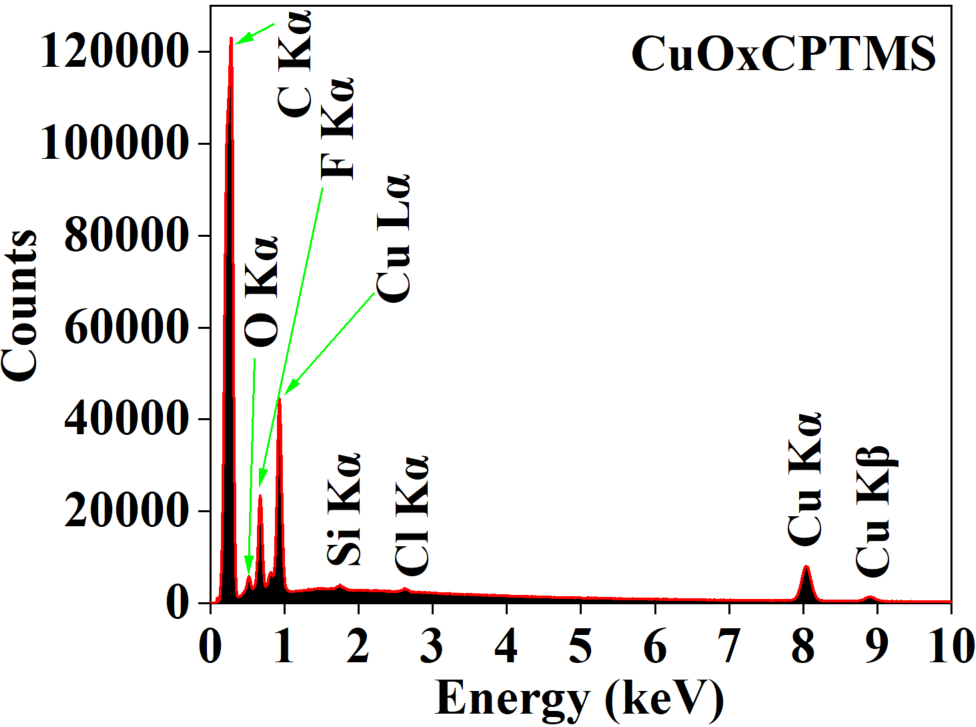


**Figure S3.** EDS of CuO*_x_*CPTMS.


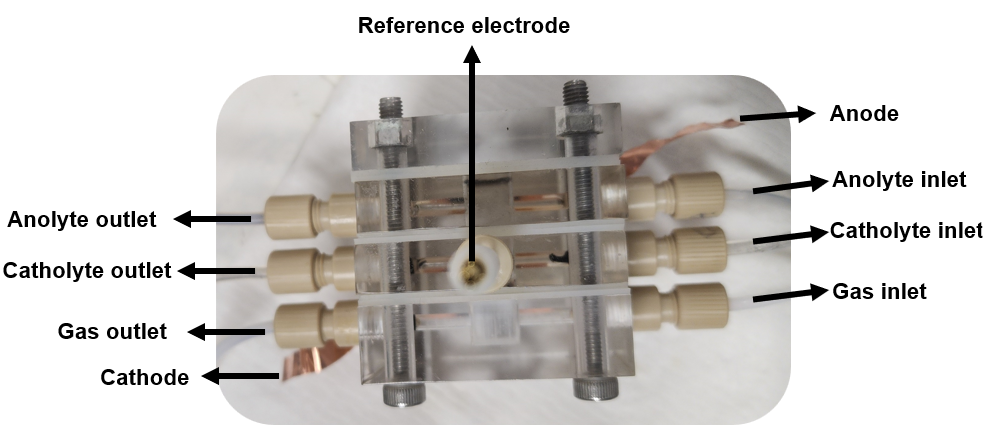


**Figure S4.** Configuration of electrochemical flow-cell for CO_2_RR.^[15]^


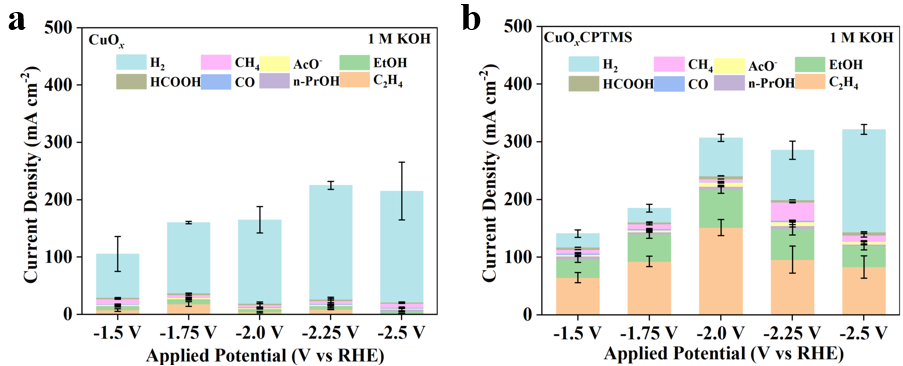


**Figure S5.** Current density during CO_2_RR in 1 M KOH of (a) CuO*_x_*, and (b) CuO*_x_*CPTMS.


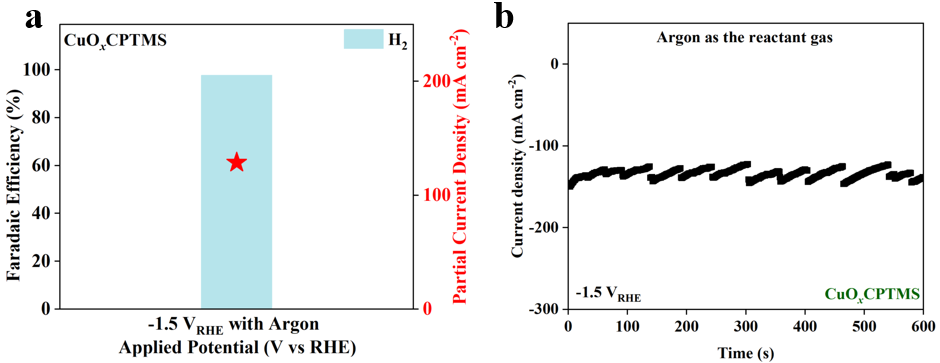


**Figure S6.** (a) Performance test result of CuO*_x_*CTPMS in Argon environment at -1.5 V_RHE_, and (b) Current-Time curve of CuO_x_CPTMS during reduction of Argon at -1.5 V_RHE._


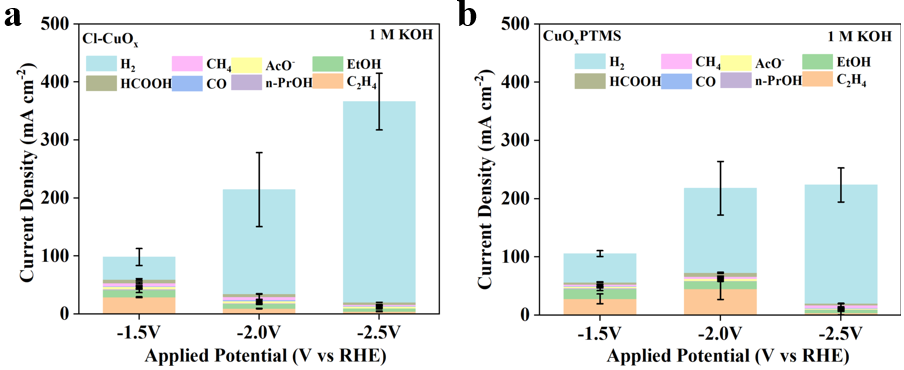


**Figure S7.** Current density during CO_2_RR in 1 M KOH of (a) Cl-CuO*_x_*, and (b) CuO*_x_*PTMS.


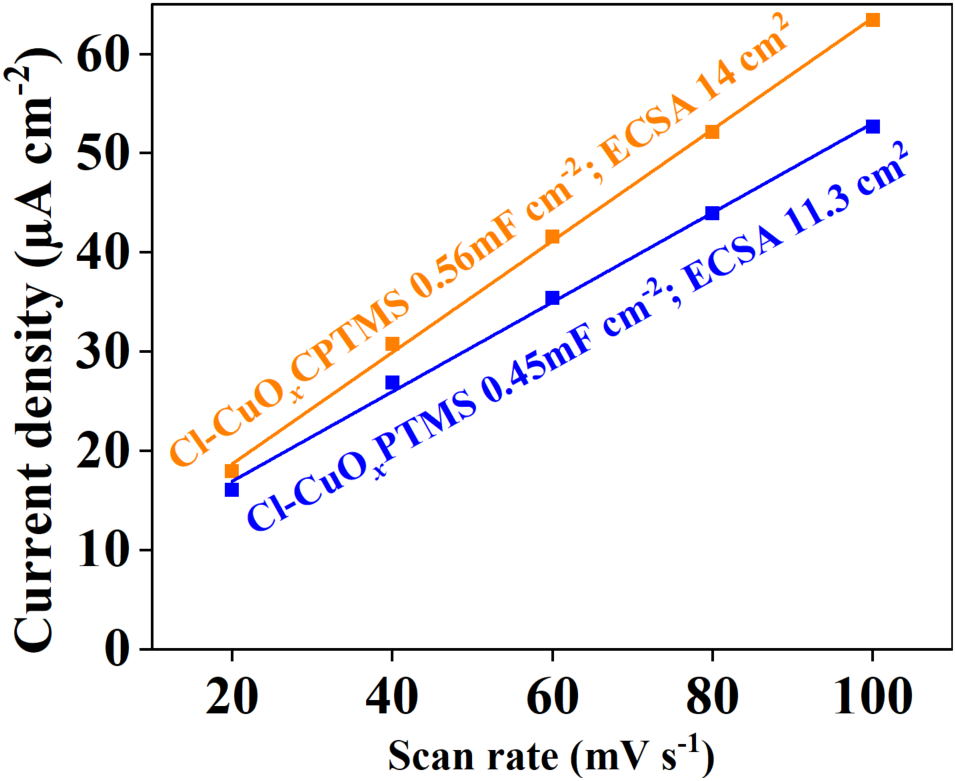


**Figure S8.** ECSA values of Cl-CuO*_x_*PTMS and Cl-CuO*_x_*CPTMS.


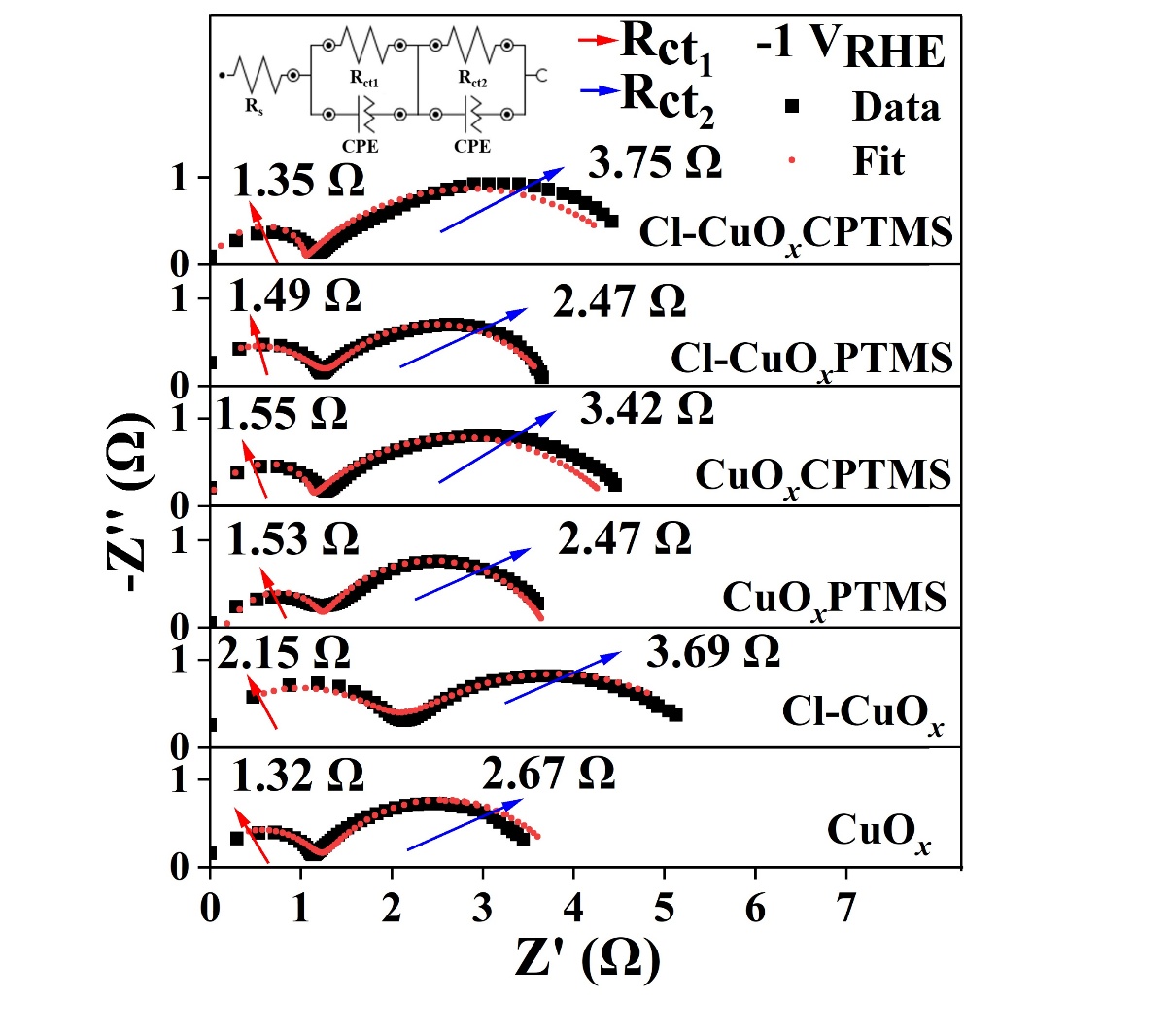


**Figure S9.** EIS of the catalysts.


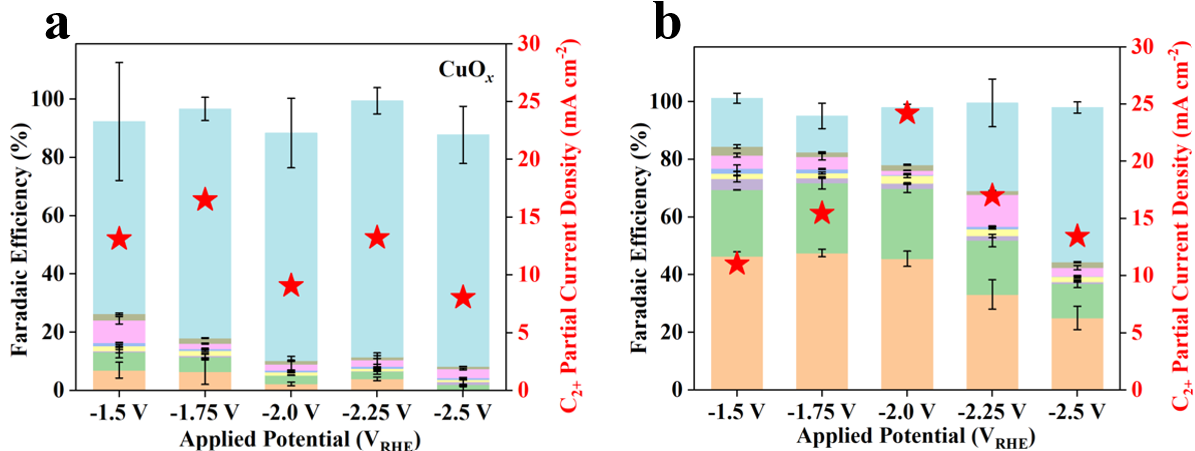


**Figure S10.** Faradaic efficiency and the associated ECSA normalized C_2+_ products partial current density during CO_2_RR in 1 M KOH of (a) CuO*_x,_* and (b) CuO*_x_*CPTMS.


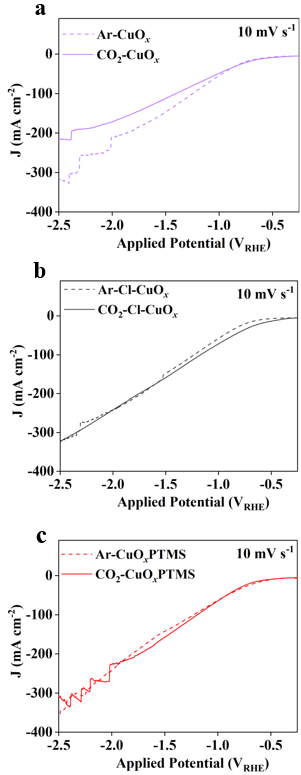


**Figure S11.** iR drop uncompensated LSV in Ar and CO_2_ environments of (a) CuO*_x_*_,_ (b) Cl-CuO*_x_*_,_ and (c) CuO*_x_*PTMS


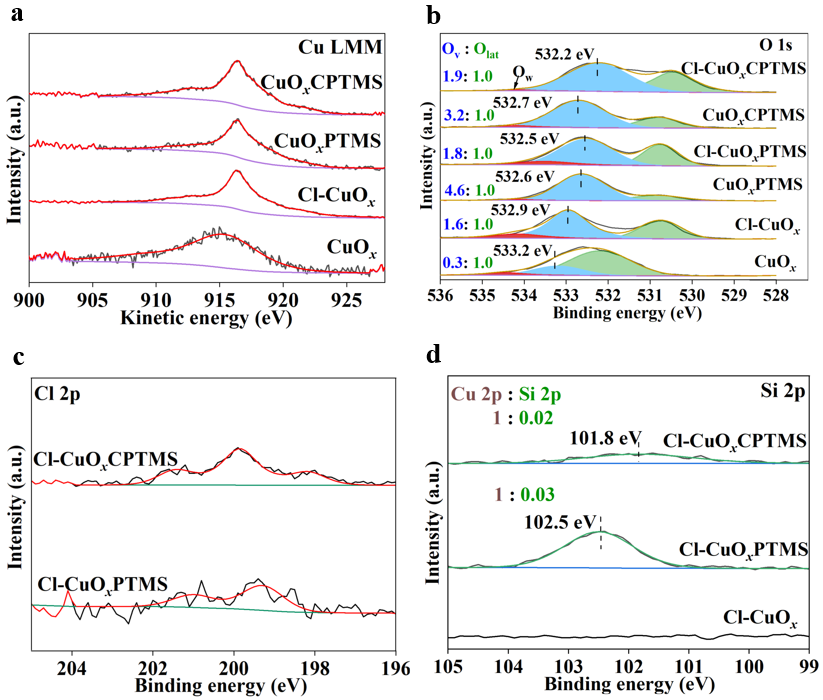


**Figure S12.** (a) CuLMM Auger, (b) O 1s XPS, (c) Cl 2p XPS, and (d) Si 2p XPS.


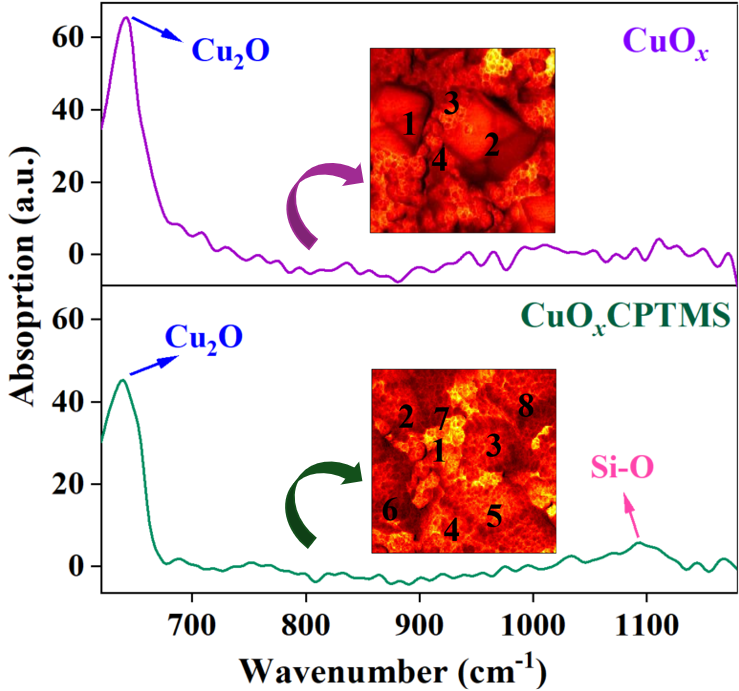


**Figure S13.** Averaged Nano-IR* spectra of CuO*_x_* and CuO*_x_*CPTMS; nano-FTIR images and the corresponding probed areas as insets (4 probed sites on CuO*_x_*; 8 probed sites on CuO*_x_*CPTMS).

* Nano-IR measurements were conducted using a nea-SCOPE system (commercially available from attocube.com/neaspec), which integrates an atomic force microscopy (AFM)-based scattering-type scanning near-field optical microscopy (s-SNOM) with a broadband illumination source. A platinum–iridium-coated metallic tip is illuminated by a coherent difference frequency generation laser (Toptica Photonica AG, Gräfelfing, Germany) that spans a spectral range from 600 to 1300 cm^−1^ and delivers ca. 500 µW of power. The back-scattered light resulting from the tip-sample interaction is detected interferometrically, using an asymmetric Michelson interferometer, at a liquid nitrogen-cooled mercury–cadmium–telluride (MCT) detector. This configuration enables the extraction of complex phase and amplitude values corresponding to vibrational absorption and reflection. The probe tip is oscillated at its fundamental resonance frequency (Ω) and demodulated at higher harmonics (nΩ) to minimize background contributions, effectively isolating the near-field signal that encodes local material information. All nano-IR spectra were collected with spectral resolution of 10 cm^-1^ at a probing depth of 15 nm.


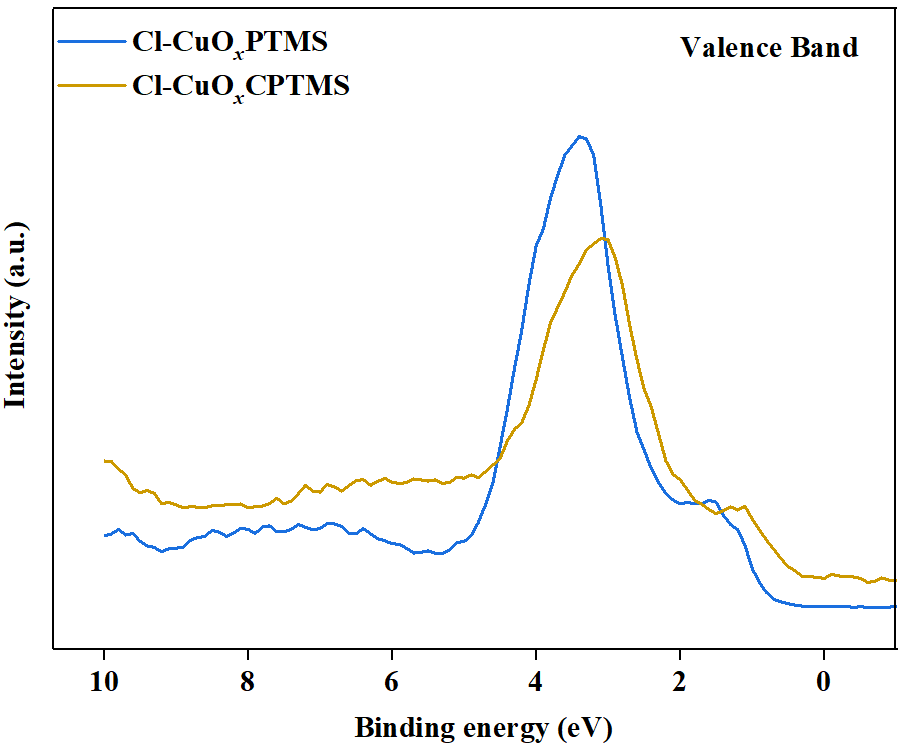


**Figure S14.** Valence band spectra of Cl-CuO*_x_*PTMS and Cl-CuO*_x_*CPTMS.


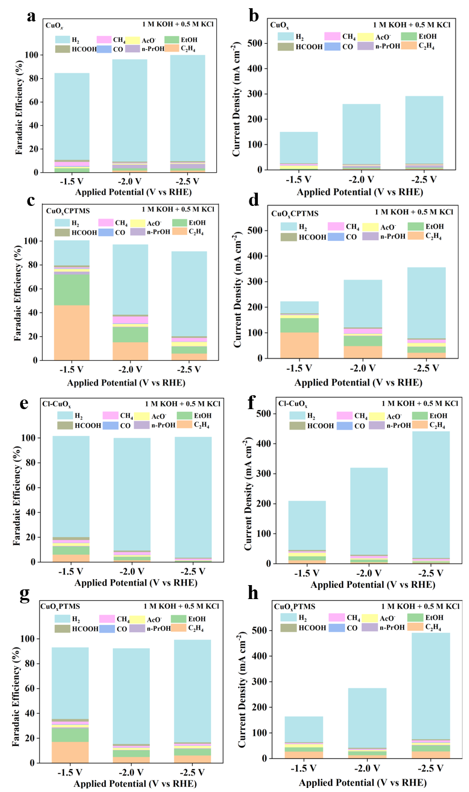


**Figure S15.** Faradaic efficiency and the associated current density during CO_2_RR in 1 M KOH + 0.5 M KCl of (a-b) CuO*_x_*, (c-d) CuO*_x_*CPTMS, (e-f) Cl-CuO*_x_*, and (g-h) CuO*_x_*PTMS.

**
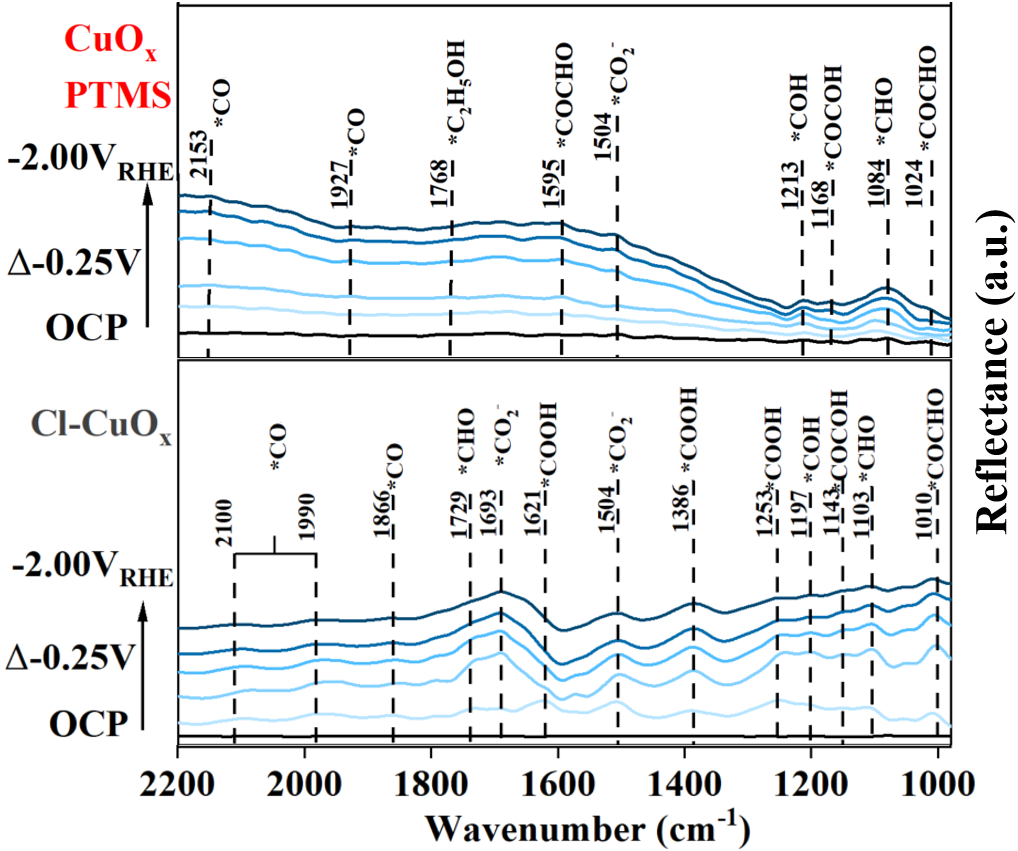
**

**Figure S16.** *In-situ* SR-FTIR spectra recorded during CO_2_RR on the surfaces of Cl-CuO*_x_* (top) and CuO*_x_*PTMS (bottom).


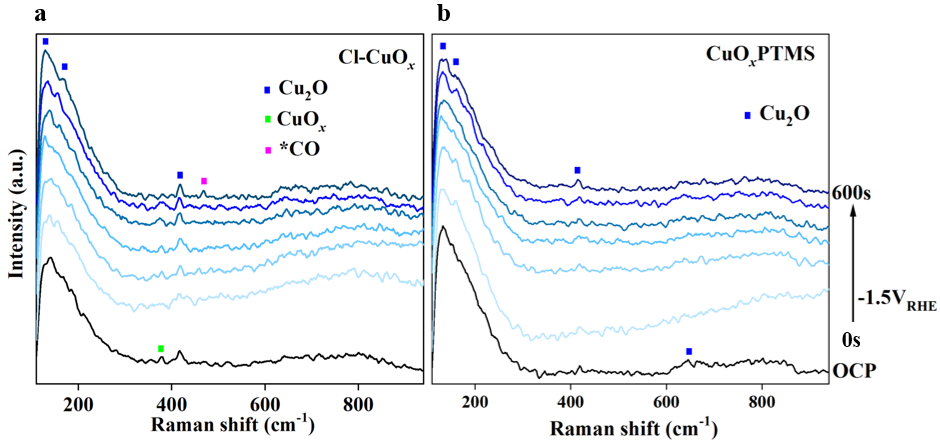


**Figure S17.** *In-situ* Raman spectra recorded at negative applied potentials on (a) Cl-CuO*_x_*, and (b) CuO*_x_*PTMS.


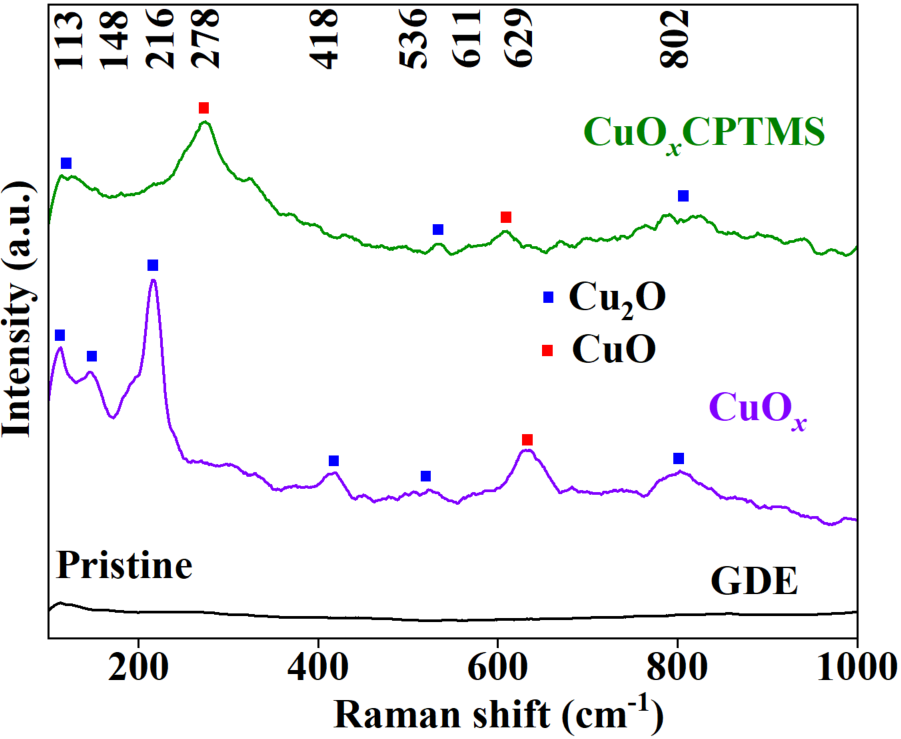


**Figure S18.** *Ex-situ* Raman spectroscopy of CuO*_x_* and CuO*_x_*CPTMS.


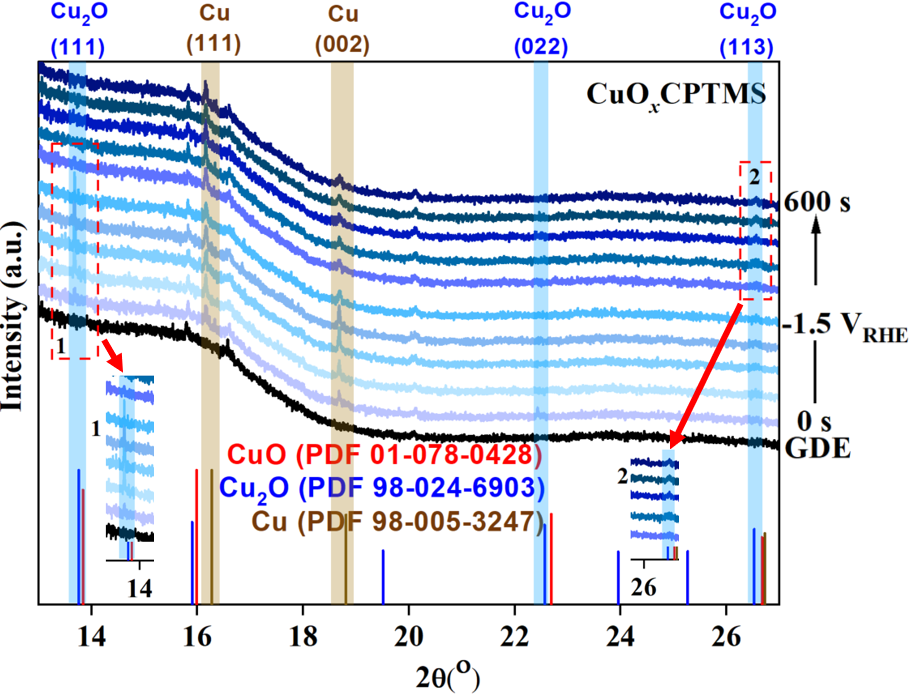


**Figure S19.** *In-situ* SR-XRPD (at 0.5904 Å) patterns of CuO*_x_*CPTMS.

**
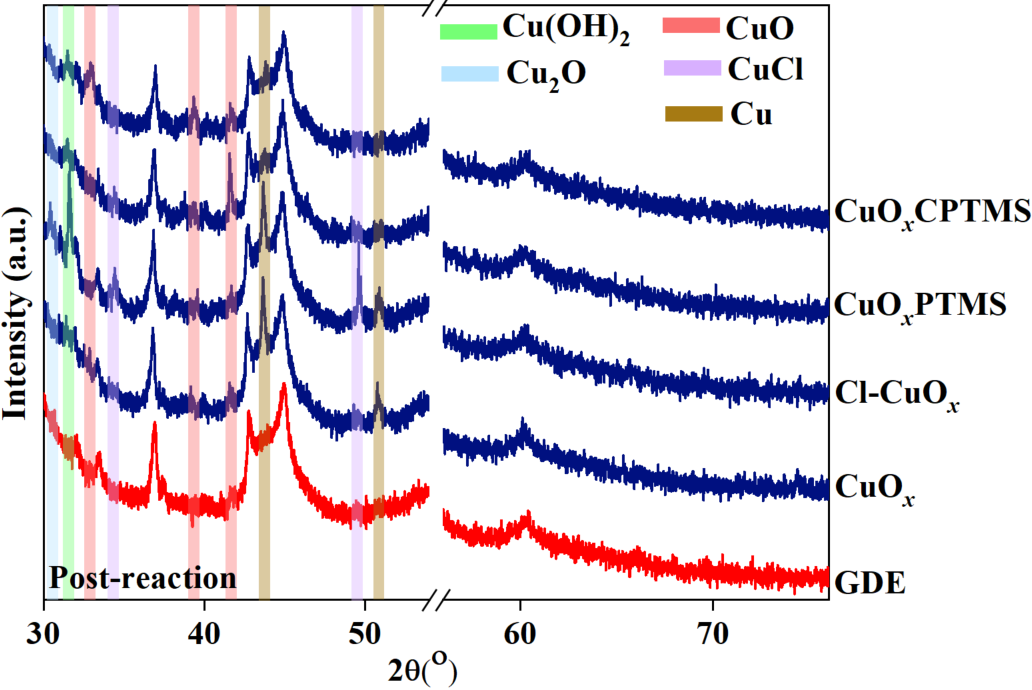
**

**Figure S20.** *Ex-situ* XRD post reaction of CuO*_x_*_,_, Cl-CuO*_x_*, CuO*_x_*PTMS, and CuO*_x_*CPTMS

**
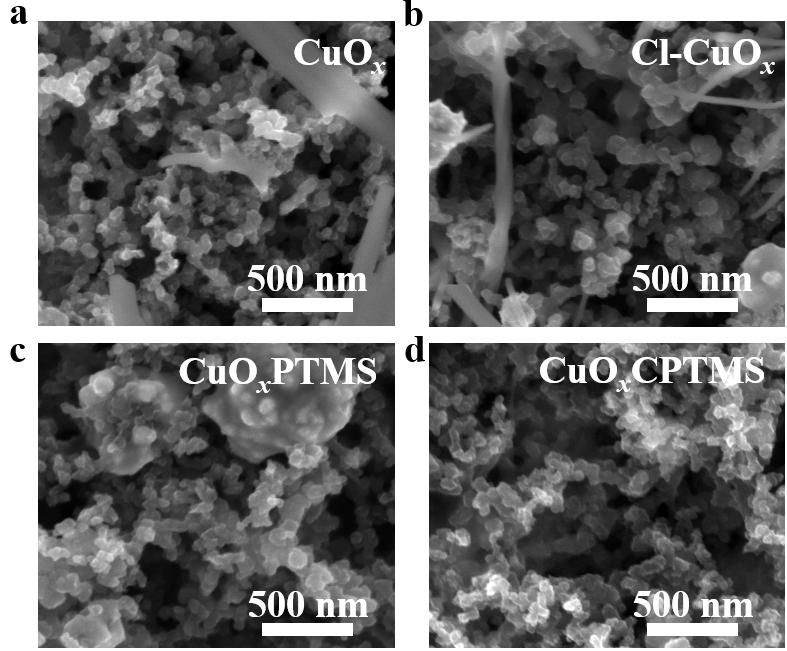
**

**Figure S21.** SEM images post reaction of (a) CuO*_x_*, (b) Cl-CuO*_x_*, (c) CuO*_x_*PTMS, and (d) CuO*_x_*CPTMS


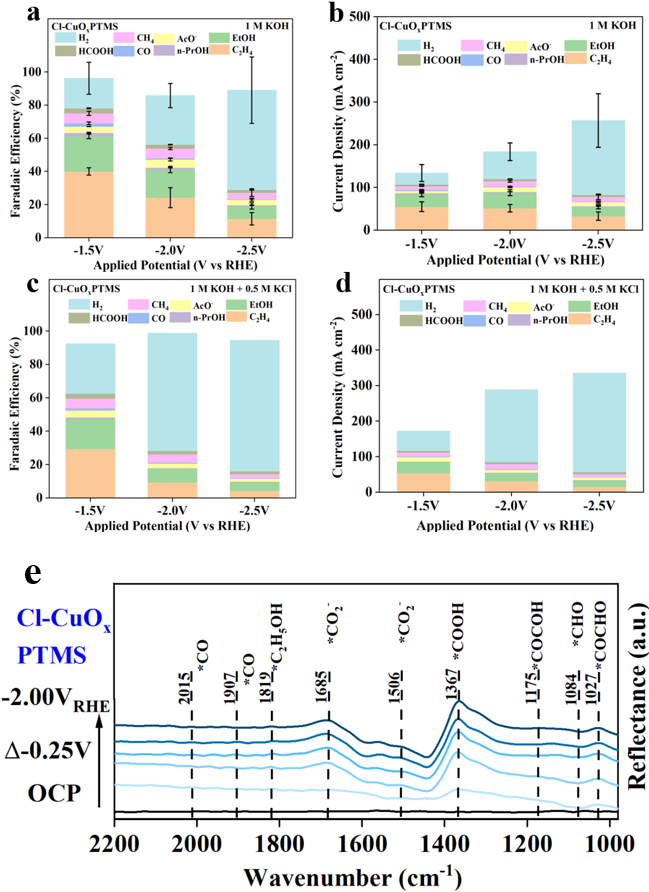


**Figure S22.** Faradaic efficiency and the associated current density of Cl-CuO*_x_*PTMS (a-b) during CO_2_RR in 1 M KOH, (c-d) during CO_2_RR in 1 M KOH + 0.5 M KCl, and (e) *in-situ* SR-FTIR spectra recorded during CO_2_RR of Cl-CuO*_x_*PTMS

**
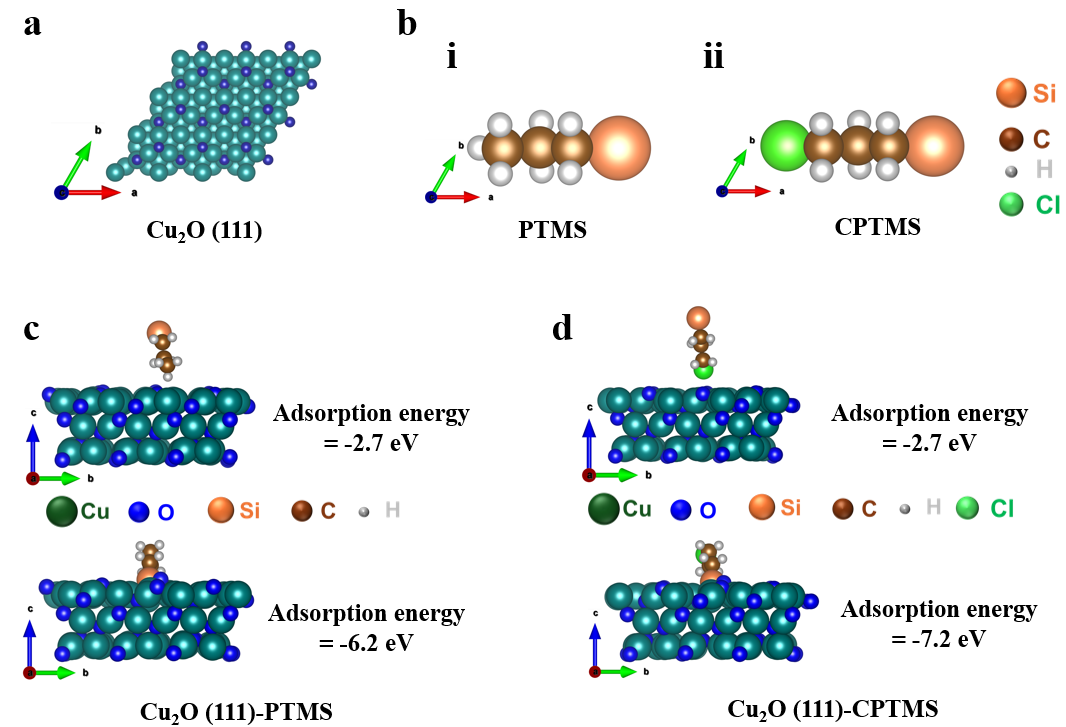
**

**Figure S23.** (a) DFT model on Cu_2_O (111), (b) DFT models of (i) PTMS and (ii) CPTMS, (c) DFT models of Cu_2_O (111)–PTMS, and (d) DFT models of Cu_2_O (111)–CPTMS


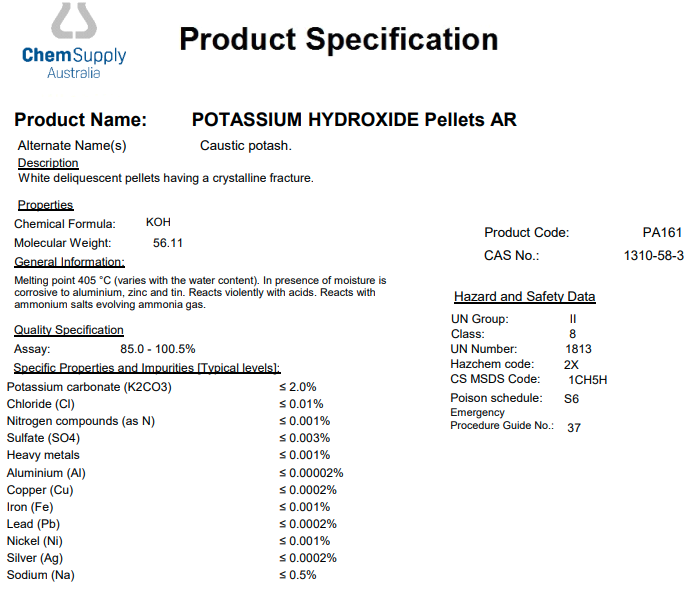


**Figure S24**. Certificate of analysis of 90% KOH from Chem-Supply


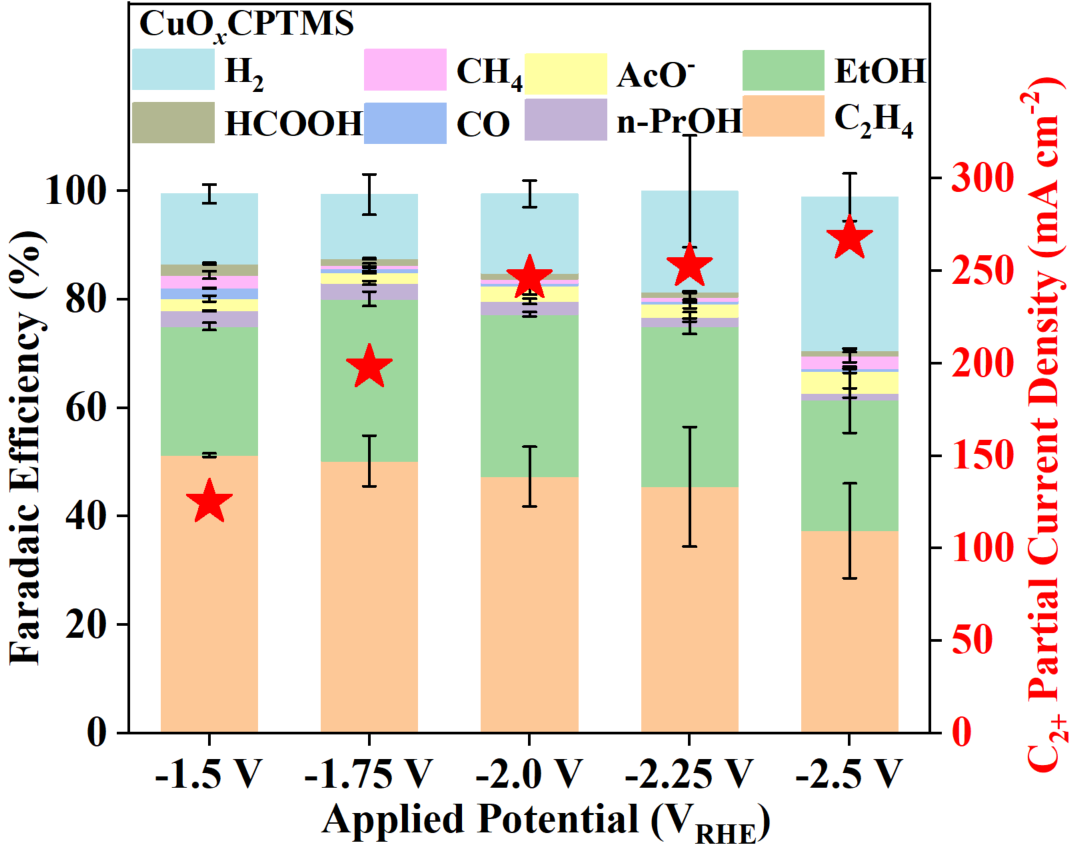


**Figure S25.** CO_2_RR performance of using 99.98% KOH on CuO*_x_*CPTMS*

*The results show a slight improvement in the C_2+_ selectivity at a higher current density due to the higher alkalinity and higher ions concentration from 99.98% KOH.^[16,17]^ However, the selectivity on products distribution confirmed the validity of our reported CO_2_RR performance *i.e.* these impurities are unlikely to affect the final performance.

**
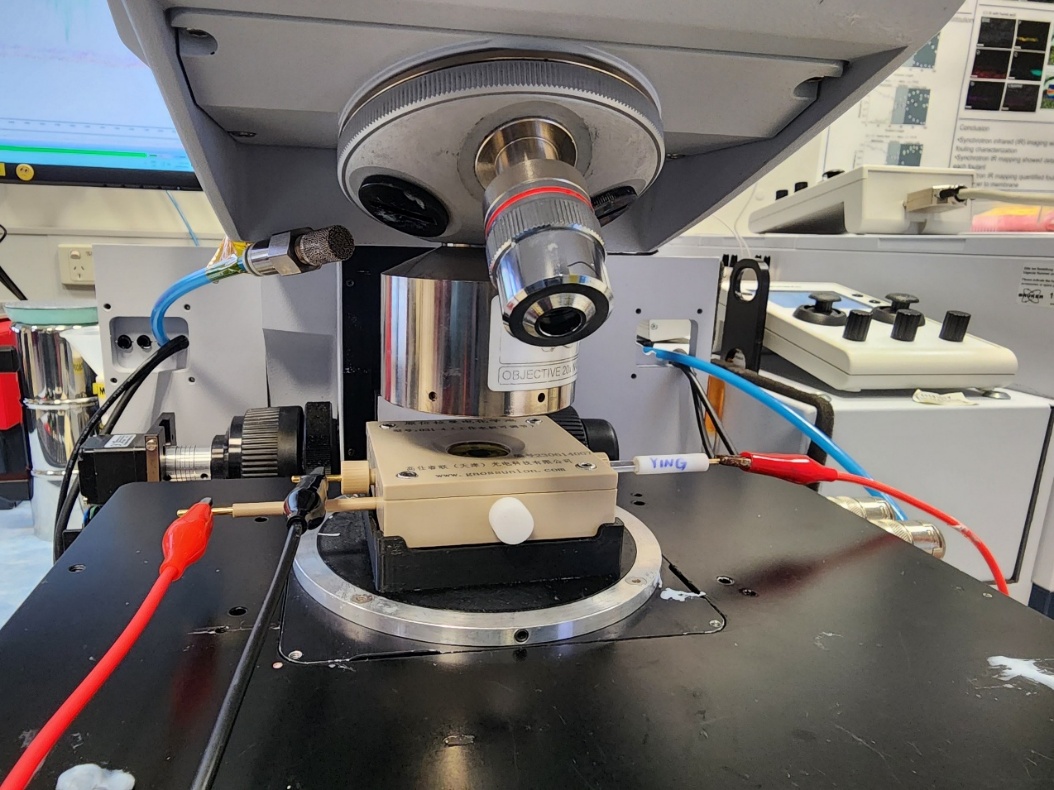
**

**Figure S26.** *In-situ* SR-FTIR setup in reflectance mode and the reaction cell. Note that the same reaction cell was used for the acquisition of *in-situ* Raman spectra.


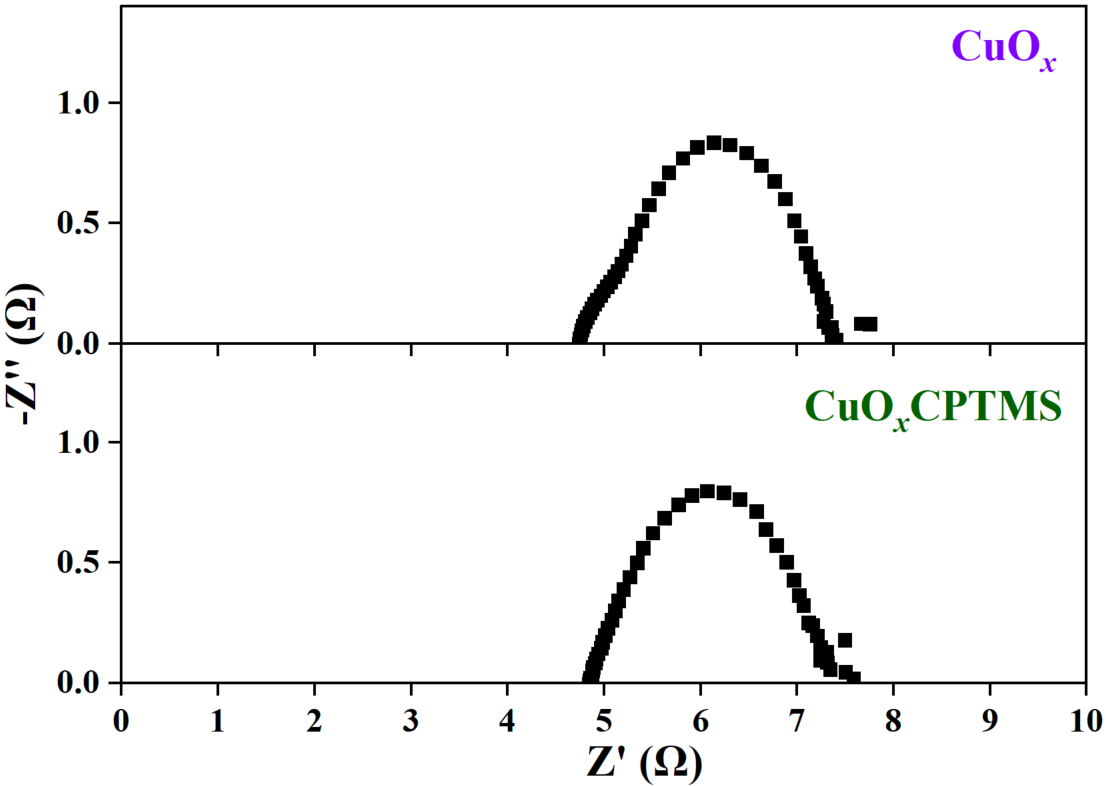


**Figure S27**. EIS of CuO*_x_* and CuO*_x_*CPTMS including the Ohmic resistance.

**
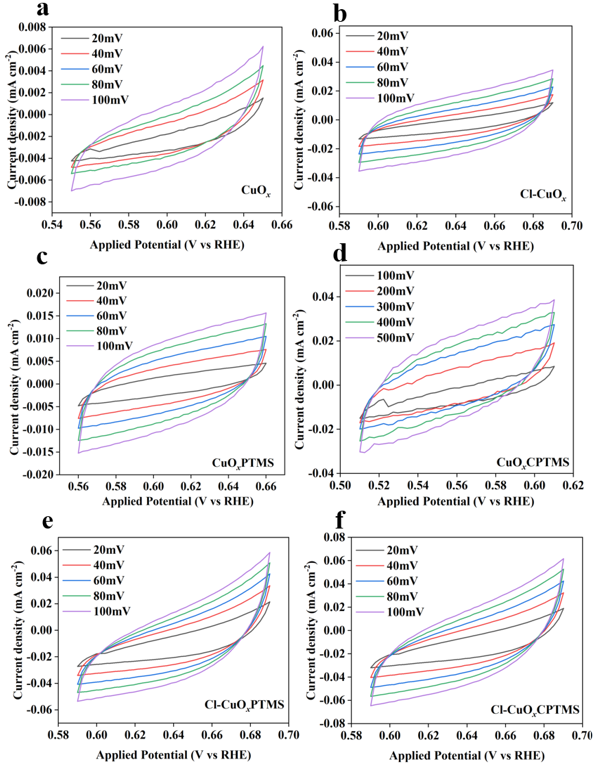
**

**Figure S28.** Cyclic voltammetry of (a) CuO*_x_*, (b) Cl-CuO*_x_*, (c) CuO*_x_*PTMS, (d) CuO*_x_*CPTMS, (e) Cl-CuO*_x_*PTMS, and (f) Cl-CuO*_x_*CPTMS

**3. Supplementary Tables**

**Table S1.** Copper based catalysts modification with halide or Si, their respective CO_2_RR performance and the contribution from the modifications

| Year | Modification | Synthesis Method | Electrolyte | C2+, CO2RR; Partial J | Coupling | Cu Species | Ref |
| --- | --- | --- | --- | --- | --- | --- | --- |
| 2025 | Br | Hydrothermal | 0.2 M K_2_SO_4_ | 72.1 C_2+_, C_2_H_4_ 47.4%; 60% C_2+_ 10h; 222 mA cm^-2^ | *CO + *CHO | Cu/Cu^+^ | ^[18]^ |
| 2025 | I | Solvothermal | 0.1 M CsI | 73.1% C_2_H_4_; 32.3 mA cm^-2^ | *CO+*CO to *COCOH; low *COOH energy barrier | Cu^2+^/Cu^+^ | ^[19]^ |
| 2025 | Br | electroanodization + annealing | 0.5 M KHCO_3_ | 98% CO at -0.4V; 42% C_2_H_6_ or 60% C_2+_ at -0.6V | enhanced CO_2_ adsorption and *COOH formation to *COCO and *COCHO | Cu^+^/Cu^0^ | ^[20]^ |
| 2025 | I | successive ion layer adsorption and reaction (SILAR) | 1M KOH + 0.05 M KI | 75.8% C_2+_; 100 mA cm^-2^ |  | Cu^2+^/Cu^+^/Cu^0^ | ^[21]^ |
| 2025 | Cl | one pot synthesis | 0.1 M KHCO_3_ + 0.2 M KCl | 40% C_2_H_4_; 80 mA cm^-2^ | *OCCHO | Cu^+^/Cu^0^ | ^[22]^ |
| 2025 | I | high temperature oxidation up to 500 ºC | 1 M KOH | 69.7% C_2+_; 279 mA cm^-2^, 57.4% C_2_H_4_; 230mA cm^-2^ | not available; only mentioned C-C coupling | Cu^+^/Cu^0^ | ^[23]^ |
| 2025 | I | one pot synthesis + electroreduction | 1 M KOH | 79% C_2+_; 237 mA cm^-2^ | *CO+*CHO |  | ^[24]^ |
| 2025 | Si | hydrothermal | 1 M KOH | 33.1% C_2_H_4_; 66 mA cm^-2^ | *CO+*CHO |  | ^[25]^ |
| 2025 | Si | one pot synthesis + calcination | 0.1 M KHCO_3_ | 42% C_2_H_4_; 6.3 mA cm^-2^ | *COCHO |  | ^[26]^ |
| 2024 | Si | wet chemical + calcination | 1 M KOH | 81.9% C_2+_; 82 mA cm^-2^ | *OCCO |  | ^[27]^ |
| 2024 | Si | wet chemical + high temperature reduction | 2 M KOH | 56% C_2_H_4_; 212 mA cm^-2^ | *COCHO |  | ^[28]^ |
|  | Cl + Si | **One-step** Electrodeposition | 1 M KOH | C_2+_ 75%, 105 mA cm^-2^; C_2_H_4_ 46%, (-1.5 V_RHE_); C_2+_ 74%, 230 mA cm^-2^; C_2_H_4_ 46% (-2.0 V_RHE_) |  |  |  |
|  | Cl + Si | **One-step** Electrodeposition | 1 M KOH + 0.5 M KCl | C_2+_ 77%, 171 mA cm^-2^; C_2_H_4_ 46%, 102 mA cm^-2^ |  |  |  |

**Table S2.** Concentration of Si and Cl during electrodeposition and the resultant ECSA values

| Catalyst | Electrodeposition bath | | ECSA (cm^2^) |
| --- | --- | --- | --- |
|  | Si Concentration | Cl Concentration |  |
| CuO*_x_* | 0 mM | 0 mM | 1.3 |
| Cl-CuO*_x_* | 0 mM | 5 mM | 10.3 |
| CuO*_x_*PTMS | 2.7 mM | 0 mM | 4.8 |
| Cl-CuO*_x_*PTMS | 2.7 mM | 5 mM | 11.3 |
| CuO*_x_*CPTMS | 2.7 mM | 2.7 mM | 9.5 |
| Cl-CuO*_x_*CPTMS | 2.7 mM | 7.7 mM | 14 |

**Table S3.** Concentration of Si and Cl during electrodeposition and the resultant calculated *d-*band center based on XPS valence band (VB)

| Catalyst | Electrodeposition in 5mM CuSO_4_. 5H_2_O | | calculated *d*-band centre from XPS VB spectra (weighted average)^[29]^ |
| --- | --- | --- | --- |
|  | Cl (mM) | Si (mM) |  |
| CuO*_x_* | 0.0 | 0.0 | 5.42 |
| Cl-CuO*_x_* | 5.0 | 0.0 | 4.75 |
| CuO*_x_*PTMS | 0.0 | 2.7 | 5.20 |
| Cl-CuO*_x_*PTMS | 5.0 | 2.7 | 4.49 |
| CuO*_x_*CPTMS | 2.7 | 2.7 | 4.96 |
| Cl-CuO*_x_*CPTMS | 7.7 | 2.7 | 4.72 |

**Table S4.** FTIR spectra band assignment for intermediates during CO_2_RR

| Wavenumber (cm^-1^) | Assignment (s) | Literature |
| --- | --- | --- |
| 1022 | *COCHO | ^[30–32]^ |
| 1064 - 1054; 1729 | *CHO | ^[33–35]^ |
| 1191 - 1035 | *COH | ^[36–38]^ |
| 1203-1159 | *COCOH | ^[30–32]^ |
| 1373-1361 | *COOH | ^[38]^ |
| 1510-1504 | *CO_2_^-^ | ^[39]^ |
| 1587 - 1566 | *COCHO or *COCOH | ^[30,33,35,40–43]^ |
| 1828 | *C_2_H_5_OH | ^[30–32]^ |
| 1890 | *CO bridge | ^[41]^ |
| 2100 - 1900 | *CO atop | ^[38]^ |

**Table S5.** Integrated area from *ex-situ* SR-XRPD on CuO (-113) and from in*-situ* SR-XRPD Cu (002)

| Catalyst | Figure 1a | Figure S19 | | | |
| --- | --- | --- | --- | --- | --- |
|  | CuO (-113) area | Cu (111) area | | Cu (002) area | |
|  |  | Before Reaction | After Reaction | Before Reaction | After Reaction |
| CuO*_x_* | 441 |  |  |  |  |
| CuO*_x_*CPTMS | 266 | 711 | 728 | 448 | 484 |

**References**

[1] Z. Zhang, T. Trần-Phú, J. Yuwono, Z. Ma, Y. Yang, J. Leverett, R.K. Hocking, B. Johannessen, P. Kumar, R. Amal, R. Daiyan, Stable dual metal oxide matrix for tuning selectivity in acidic electrochemical carbon dioxide reduction, Applied Catalysis B: Environment and Energy 371 (2025): 125203, https://doi.org/10.1016/j.apcatb.2025.125203.

[2] K.P. Kuhl, E.R. Cave, D.N. Abram, T.F. Jaramillo, New insights into the electrochemical reduction of carbon dioxide on metallic copper surfaces, Energy & Environmental Science 5 (2012): 7050. https://doi.org/10.1039/c2ee21234j.

[3] G. Kresse, Ab initio molecular-dynamics simulation of the liquid-metal-amorphous-semiconductor transition in germanium, Physical Review B 8 (1994): 15–1994.

[4] G. Kresse, J. Furthmü, Efficient iterative schemes for ab initio total-energy calculations using a plane-wave basis set, Physical Review B 54 (1996): 11169.

[5] G. Kresse, D. Joubert, From ultrasoft pseudopotentials to the projector augmented-wave method, Physical Review B 59 (1999): 1758.

[6] J.P. Perdew, K. Burke, M. Ernzerhof, Generalized Gradient Approximation Made Simple, Physical Review Letters 77 (1996): 3865, https://doi.org/10.1103/PhysRevLett.77.3865.

[7] W. Kohn, L.J. Sham, Self-Consistent Equations Including Exchange and Correlation Effects, Physical Review 140 (1965): A1133, https://doi.org/10.1103/PhysRev.140.A1133.

[8] A. Jain, S.P. Ong, G. Hautier, W. Chen, W.D. Richards, S. Dacek, S. Cholia, D. Gunter, D. Skinner, G. Ceder, K.A. Persson, Commentary: The materials project: A materials genome approach to accelerating materials innovation, APL Materials 1 (2013): 011002, https://doi.org/10.1063/1.4812323.

[9] A. Hjorth Larsen, J. JØrgen Mortensen, J. Blomqvist, I.E. Castelli, R. Christensen, M. Dułak, J. Friis, M.N. Groves, B. Hammer, C. Hargus, E.D. Hermes, P.C. Jennings, P. Bjerre Jensen, J. Kermode, J.R. Kitchin, E. Leonhard Kolsbjerg, J. Kubal, K. Kaasbjerg, S. Lysgaard, J. Bergmann Maronsson, T. Maxson, T. Olsen, L. Pastewka, A. Peterson, C. Rostgaard, J. SchiØtz, O. Schütt, M. Strange, K.S. Thygesen, T. Vegge, L. Vilhelmsen, M. Walter, Z. Zeng, K.W. Jacobsen, The atomic simulation environment—a Python library for working with atoms, Journal of Physics: Condensed Matter 29 (2017): 273002, https://doi.org/10.1088/1361-648X/aa680e.

[10] S.L. Dudarev, G.A. Botton, S.Y. Savrasov, C.J. Humphreys, A.P. Sutton, Electron-energy-loss spectra and the structural stability of nickel oxide:  An LSDA+U study, Physical. Review B 57 (1998): 1505, https://doi.org/10.1103/PhysRevB.57.1505.

[11] A.K. Mishra, A. Roldan, N.H. De Leeuw, A density functional theory study of the adsorption behaviour of CO2 on Cu2O surfaces, Journal of Chemical Physics 145 (2016): 44709, https://doi.org/10.1063/1.4958804.

[12] S. Kim, J. Chen, T. Cheng, A. Gindulyte, J. He, S. He, Q. Li, B.A. Shoemaker, P.A. Thiessen, B. Yu, L. Zaslavsky, J. Zhang, E.E. Bolton, PubChem 2025 update, Nucleic Acids Research 53 (2025): D1516–D1525. https://doi.org/10.1093/nar/gkae1059.

[13] S. Grimme, S. Ehrlich, L. Goerigk, Effect of the damping function in dispersion corrected density functional theory, Journal of Computational Chemistry 32 (2011): 1456–1465. https://doi.org/10.1002/jcc.21759.

[14] D. Sasaki, K. Hiyoshi, W. Tang, E. Sanville, G. Henkelman, A grid-based Bader analysis algorithm without lattice bias You may also like Experiments on Distributions of Cycle Degradations of Li-Ion Batteries A grid-based Bader analysis algorithm without lattice bias, Journal of Physics: Condensed Matter 21 (2009): 7, https://doi.org/10.1088/0953-8984/21/8/084204.

[15] Y.Y. Ch’ng, Z. Ma, M. Zhang, S. Zhou, V. Kundi, P. Vijaya Kumar, W. Zhong, Y. Yang, J. Vongsvivut, Q. Gu, X. Luo, L. Jiang, R. Daiyan, Z. Han, R. Amal, Nucleation-Controlled Reconstruction of CuO x for Selective CO 2 Electroreduction, 0 (2026), https://doi.org/10.1002/adfm.202529118.

[16] C.M. Gabardo, A. Seifitokaldani, J.P. Edwards, C.-T. Dinh, T. Burdyny, M.G. Kibria, C.P. O’brien, E.H. Sargent, D. Sinton, Combined high alkalinity and pressurization enable efficient CO 2 electroreduction to CO, Energy Environ. Sci 11 (2018): 2531, https://doi.org/10.1039/c8ee01684d.

[17] H. Xiang, S. Rasul, K. Scott, J. Portoles, P. Cumpson, E.H. Yu, Enhanced selectivity of carbonaceous products from electrochemical reduction of CO2 in aqueous media, Journal of CO2 Utilization 30 (2019): 214–221, https://doi.org/10.1016/J.JCOU.2019.02.007.

[18] Y. Jiang, H. He, D. Xia, H. Zhang, S. Tan, L. Luo, Y. Kong, L. Deng, Y.-N. Liu, Halide-ion induced reconstruction of Cu-based catalysts to form stable Cu 0 /Cu + sites enabling efficient electroreduction of CO 2 to C 2+ products, Green Chemistry 27 (2025): 9737, https://doi.org/10.1039/d5gc03114a.

[19] Y. Peng, Y. Chen, Z. Wang, Y. Yin, J. Li, H. Liu, J. Jiao, W. Zhao, R. Duan, P. Zhang, J. Yang, H. Wang, X. Xing, Z. Liu, S. Yang, X. Kang, B. Han, Selective electroreduction of carbon dioxide to ethylene over stable iodide-induced asymmetric copper sites within a metal-organic polyhedron, Science Bulletin 70 (2025): 2641–2649, https://doi.org/10.1016/j.scib.2025.06.001.

[20] Z. Lv, G. Ma, H. Mu, J. Guo, M. Zhu, J. Li, F. Li, Mechanistic insights into the impact of bromide ion adsorption and surface bromination on Cu2O for enhanced selectivity and activity in electrochemical CO2 reduction, Journal of Colloid And Interface Science 692 (2025): 137492, https://doi.org/10.1016/j.jcis.2025.137492.

[21] F.N.I. Sari, T.H. Wang, M.C. Hsieh, T.H. Kuan, M.A. Camargo-Valero, C.Y. Lin, Iodide-mediated electroreduction of carbon dioxide for efficient and selective electrosynthesis of multicarbon products over copper iodide microcrystals, Journal of Colloid And Interface Science 696 (2025): 137847, https://doi.org/10.1016/j.jcis.2025.137847.

[22] B. Chen, W. Li, R. Xia, X. Wang, H. Hu, H. Dai, Q. Guan, C. Wang, Y. Liu, W. Li, Constructing and stabilizing Cu0/Cu+ sites through dual chlorine-induced strategy for CO2 electroreduction to C2H4, Journal of Colloid And Interface Science 699 (2025): 138237, https://doi.org/10.1016/j.jcis.2025.138237.

[23] H. Wang, Q. Wu, R. Du, G. Chen, Tuning *CO Adsorption via Cu + /Cu 0 Interface Engineering for Enhanced Ethylene Selectivity in Electrochemical CO 2 Reduction, ACS Applied Materials & Interfaces 17 (2025): 55003–55012. https://doi.org/10.1021/acsami.5c15459.

[24] Z. Yan, M. Liu, Z. Guo, Q. Chen, Z. Xi, X.-Z. Sun, J. Yu, T. Wu, Z. Yan, M. Liu, Z. Guo, Q. Chen, Z. Xi, T. Wu, X.-Z. Sun, Trace Iodine Modified Copper Catalyst Drives Asymmetric C─C Coupling in Stable CO 2 Electroreduction, Advanced Functional Materials 35 (2025): 2420493, https://doi.org/10.1002/adfm.202420493.

[25] S. Yan, S. Gong, S. Zhang, H. Sun, H. Yu, L. Chen, J. Han, H. Wang, In Situ/Operando Insights into the Selectivity of CH4/C2H4 in CO2 Electroreduction by Fine-Tuning the Composition of Cu/SiO2 Catalysts, ChemSusChem 18 (2025): 202402461, https://doi.org/10.1002/cssc.202402461.

[26] X. Ji, W. Xu, C. Liu, X. Wang, CuO doping SiO2 for enhancing electron transfer to product C2H4 in electrocatalytic CO2 reduction, Electrochimica. Acta 525 (2025): 146090, https://doi.org/10.1016/j.electacta.2025.146090.

[27] L. Cheng, R. Wang, W. Si, Y. Deng, J. Li, Y. Peng, Enhancing CO2 Electroreduction Performance through Si-Doped CuO: Stabilization of Cu+/Cu0 Sites and Improved C2 Product Selectivity, ACS Catalysis 14 (2024): 10324–10333, https://doi.org/10.1021/acscatal.4c01961.

[28] W.-F. Xiong, D.-H. Si, H.-F. Li, X. Song, T. Wang, Y.-B. Huang, T.-F. Liu, T. Zhang, R. Cao, Steering CO 2 Electroreduction Selectivity U-Turn to Ethylene by Cu−Si Bonded Interface, Journal of the American Chemical Society 146 (2024): 289–297, https://doi.org/10.1021/jacs.3c08867.

[29] H.K. Wang, C.Y. Sit, R.T. Haasch, P.J.A. Kenis, A.A. Gewirth, Effect of Polymer Variation in Cu-Polymer Composite Electrodes on Electrochemical CO2 Reduction, ACS Applied Energy Materials 8 (2025): 2993–3002, https://doi.org/10.1021/acsaem.4c03092.

[30] R. Du, Q. Wu, S. Zhang, P. Wang, Z. Li, Y. Qiu, K. Yan, G.I.N. Waterhouse, P. Wang, J. Li, Y. Zhao, W.W. Zhao, X. Wang, G. Chen, Cu-C(O) Interfaces Deliver Remarkable Selectivity and Stability for CO2 Reduction to C2+ Products at Industrial Current Density of 500 mA cm−2, Small 19 (2023): 2301289, https://doi.org/10.1002/smll.202301289.

[31] Q. Liu, H. Cheng, T. Chen, T.W.B. Lo, Z. Xiang, F. Wang, Regulating the ∗OCCHO intermediate pathway towards highly selective photocatalytic CO2reduction to CH3CHO over locally crystallized carbon nitride, Energy Environ. Sci. 15 (2022): 225–233, https://doi.org/10.1039/d1ee02073k.

[32] J.-Y. Ye, Y.-X. Jiang, T. Sheng, S.-G. Sun, In-situ FTIR spectroscopic studies of electrocatalytic reactions and processes, Nano Energy 29 (2016): 414–427, https://doi.org/10.1016/j.nanoen.2016.06.023.

[33] X.F. Qiu, H.L. Zhu, J.R. Huang, P.Q. Liao, X.M. Chen, Highly Selective CO2 Electroreduction to C2H4Using a Metal-Organic Framework with Dual Active Sites, J. Am. Chem. Soc. 143 (2021): 7242–7246, https://doi.org/10.1021/jacs.1c01466.

[34] X. Zhu, H. Xu, J. Liu, C. Bi, J. Tian, K. Zhong, B. Wang, P. Ding, X. Wang, P.K. Chu, H. Xu, J. Ding, Stacking Engineering of Heterojunctions in Half-Metallic Carbon Nitride for Efficient CO2 Photoreduction, Advanced Science 10 (2023): 202307192, https://doi.org/10.1002/advs.202307192.

[35] T. Zhang, B. Yuan, W. Wang, J. He, X. Xiang, Tailoring *H Intermediate Coverage on the CuAl2O4/CuO Catalyst for Enhanced Electrocatalytic CO2 Reduction to Ethanol, Angew. Chem. Int. Ed. 62 (2023): 202303096, https://doi.org/10.1002/anie.202302096.

[36] Y.-R. Wang, H.-M. Ding, M. Liu, Y.-L. Yang, Y. ifa Chen, S.-L. Li, Y.-Q. Lan, Imparting CO 2 Electroreduction Auxiliary for Integrated Morphology Tuning and Performance Boosting in a Porphyrin-based Covalent Organic Framework, (2022): 202114648, https://doi.org/10.1002/anie.202114648.

[37] W. Wang, C. Deng, S. Xie, Y. Li, W. Zhang, H. Sheng, C. Chen, J. Zhao, Photocatalytic C−C Coupling from Carbon Dioxide Reduction on Copper Oxide with Mixed-Valence Copper(I)/Copper(II), Cite This: J. Am. Chem. Soc 143 (2021): 2984–2993, https://doi.org/10.1021/jacs.1c00206.

[38] S. Zhu, T. Li, W. Bin Cai, M. Shao, CO2 Electrochemical Reduction As Probed through Infrared Spectroscopy, ACS Energy Lett. 4 (2019): 682–689, https://doi.org/10.1021/acsenergylett.8b02525.

[39] W. Shangguan, Q. Liu, Y. Wang, N. Sun, Y. Liu, R. Zhao, Y. Li, C. Wang, J. Zhao, Molecular-level insight into photocatalytic CO2 reduction with H2O over Au nanoparticles by interband transitions, Nat. Commun. 13 (2022): 3894, https://doi.org/10.1038/s41467-022-31474-2.

[40] R. Zhang, H. Wang, S. Tang, C. Liu, F. Dong, H. Yue, B. Liang, Photocatalytic Oxidative Dehydrogenation of Ethane Using CO 2 as a Soft Oxidant over Pd/TiO 2 Catalysts to C 2 H 4 and Syngas, ACS Catal. 18 (2018): 54, https://doi.org/10.1021/acscatal.8b02441.

[41] Y. Yao, T. Shi, W. Chen, J. Wu, Y. Fan, Y. Liu, L. Cao, Z. Chen, A surface strategy boosting the ethylene selectivity for CO 2 reduction and in situ mechanistic insights, Nat. Commun. 15 (2024): 1257, https://doi.org/10.1038/s41467-024-45704-2.

[42] G. Li, Y. Zhao, J.P.H. Li, W. Chen, S. Li, X. Dong, Y. Song, Y. Yang, W. Wei, Y. Sun, Insight into composition and intermediate evolutions of copper-based catalysts during gas-phase co2 electroreduction to multicarbon oxygenates, Catalysts 11 (2021): 1502, https://doi.org/10.3390/catal11121502.

[43] K. Yao, J. Li, H. Wang, R. Lu, X. Yang, M. Luo, N. Wang, Z. Wang, C. Liu, T. Jing, S. Chen, E. Cortés, S.A. Maier, S. Zhang, T. Li, Y. Yu, Y. Liu, X. Kang, H. Liang, Mechanistic Insights into OC−COH Coupling in CO 2 Electroreduction on Fragmented Copper, J. Am. Chem. Soc. 144 (2022): 14005–14011, https://doi.org/10.1021/jacs.2c01044.
